# Responders and non‐responders to aerobic exercise training: beyond the evaluation of ${\dot{V}O}_{2\max}$

**DOI:** 10.14814/phy2.14951

**Published:** 2021-08-19

**Authors:** Felipe Mattioni Maturana, Rogerio N. Soares, Juan M. Murias, Philipp Schellhorn, Gunnar Erz, Christof Burgstahler, Manuel Widmann, Barbara Munz, Ansgar Thiel, Andreas M. Nieß

**Affiliations:** ^1^ Sports Medicine Department University Hospital of Tübingen Tübingen Germany; ^2^ Interfaculty Research Institute for Sport and Physical Activity Eberhard Karls University of Tübingen Tübingen Germany; ^3^ Faculty of Kinesiology University of Calgary Calgary Canada; ^4^ Institute of Sports Science Eberhard Karls University Tübingen Tübingen Germany

**Keywords:** cardiovascular, exercise training, health, responders

## Abstract

The evaluation of the maximal oxygen uptake (${\dot{V}O}_{2\max}$) following exercise training is the classical assessment of training effectiveness. Research has lacked in investigating whether individuals that do not respond to the training intervention (${\dot{V}O}_{2\max}$), also do not improve in other health‐related parameters. We aimed to investigate the cardiovascular and metabolic adaptations (i.e., performance, body composition, blood pressure, vascular function, fasting blood markers, and resting cardiac function and morphology) to exercise training among participants who showed different levels of ${\dot{V}O}_{2\max}$ responsiveness. Healthy sedentary participants engaged in a 6‐week exercise training program, three times a week. Our results showed that responders had a greater increase in peak power output, second lactate threshold, and microvascular responsiveness, whereas non‐responders had a greater increase in cycling efficiency. No statistical differences were observed in body composition, blood pressure, fasting blood parameters, and resting cardiac adaptations. In conclusion, our study showed, for the first time, that in addition to the differences in the ${\dot{V}O}_{2\max}$, a greater increase in microvascular responsiveness in responders compared to non‐responders was observed. Additionally, responders and non‐responders did not show differences in the adaptations on metabolic parameters. There is an increasing need for personalized training prescription, depending on the target clinical outcome.

## Graphical abstract

The evaluation of the maximal oxygen uptake (${\dot{V}O}_{2\max}$) following exercise training is the classical assessment of training effectiveness. Research has lacked in investigating whether individuals that do not respond to the training intervention (${\dot{V}O}_{2\max}$), also do not improve in other health‐related parameters. Our results showed that responders had a greater increase than non‐responders in peak power output, second lactate threshold, and microvascular responsiveness, whereas non‐responders had a greater increase in cycling efficiency than responders.

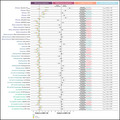

**New findings**

**What is the central question of this study?**

We aimed to answer the question of whether high responders to exercise, as characterized by changes in cardiorespiratory fitness, would also present significant changes in other health‐related variables, such as body composition, glucose control, blood lipids, and others. The central idea is that exercise is definitely not a “one‐size‐fits‐all” approach, and that current exercise guidelines are based on the general population. Understanding the interplay between the measures of fitness and measures related to cardiovascular risk would help the advance of preventive medicine and its efficacy, focusing more on the individual characteristics.

**What are the main finding and its importance?**

We showed that, in fitness‐related parameters, responders presented a greater increase in peak power output and second lactate threshold, whereas non‐responders had a greater improvement in cycling efficiency. In terms of vascular health, responders showed greater adaptations than non‐responders in microvascular responsiveness. No statistical differences were observed between responders and non‐responders in body composition, blood pressure, fasting blood parameters as well as resting cardiac adaptations.

## 1 INTRODUCTION

The efficacy of a given exercise training intervention is often evaluated by the changes in the maximal oxygen uptake (${\dot{V}O}_{2\max}$) between pre‐ and post‐training (i.e., $\Delta {\dot{V}O}_{2\max}$). In this regard, a large body of research has shown that both high‐ and moderate‐intensity training are effective in improving ${\dot{V}O}_{2\max}$ in health and disease (Mattioni Maturana et al., 2021). Although there is strong evidence that exercise training leads to increases in cardiorespiratory fitness, literature does often report “non‐responders” individuals, which are participants that do not show a significant increase in ${\dot{V}O}_{2\max}$ after a training intervention. Even though $\Delta {\dot{V}O}_{2\max}$ provides information on participants’ trainability at the individual level (Hecksteden et al., 2015, 2018; Pickering & Kiely, 2019), it is known that exercise training also improves other cardiovascular and metabolic components that may not be linked to significant changes in ${\dot{V}O}_{2\max}$ but greatly contribute to an individual's overall health (Fiuza‐Luces et al., 2013). Indeed, understanding the effects of exercise training beyond the improvement in cardiorespiratory fitness (i.e., ${\dot{V}O}_{2\max}$) becomes crucial for prescribing an effective and tailored preventive medicine strategy, avoiding the “one‐size‐fits‐all” approach in exercise prescription. To date, it still remains unknown whether individuals who show a low fitness response to given metabolic stress imposed by the exercise training, also show low responses to other parameters, such as body composition, blood lipids, and glucose and insulin metabolism. Therefore, the question arises: do non‐responders (in relation to ${\dot{V}O}_{2\max}$) to exercise training are also non‐responders to other cardiovascular and/or metabolic health endpoints? In addition, are the improvements in ${\dot{V}O}_{2\max}$ in responders accompanied by improvements in other cardiovascular and/or metabolic health parameters?

The HERITAGE study has already shown considerable inter‐individual variability in outcomes such as resting blood pressure, blood lipids, and fasting insulin even in individuals of the same family (Bouchard et al., 2012; Bouchard & Rankinen, 2001). Thus, although the beneficial effects of exercise training on cardiovascular and metabolic health seem evident (Fiuza‐Luces et al., 2013, 2018; Gabriel & Zierath, 2017; Mattioni Maturana et al., 2021), current evidence suggests that there is a multitude of factors that will influence the response from regular/chronic exercise training, such as the target endpoint (e.g., body composition, blood pressure, heart function, to cite a few), individual characteristics (e.g., population, age, sex), and training intensity (Fiuza‐Luces et al., 2018; Mattioni Maturana et al., 2021). Whether (or to what extent) maximal cardiorespiratory adaptations in response to exercise training are connected to changes in submaximal cardiovascular and metabolic responses that are essential for human health and longevity remains unknown.

Thus, the present study investigated the cardiovascular and metabolic adaptations to exercise training among participants who showed different levels of responsiveness in terms of $\Delta {\dot{V}O}_{2\max}$. Previously healthy sedentary participants engaged in a 6‐week aerobic exercise training program, which involved high‐intensity interval training (HIIT) or moderate‐intensity continuous training (MICT). Participants were then classified of responders or non‐responders based on changes in ${\dot{V}O}_{2\max}$. Specifically, we aimed to examine how responders and non‐responders adapted in other resting and submaximal exercise outcomes related to human health and performances such as peak power output, lactate thresholds, efficiency slopes, body composition, blood pressure, vascular function, fasting blood markers, and resting cardiac function and morphology. We hypothesized that participants classified as “responders” would present a greater improvement than “non‐responders” in fitness‐related measures, and “non‐responders” would still show adaptations to exercise training in other health‐related parameters.

## 2 METHODS

The current study was part of a large research project named iReAct (Individual Response to Physical Activity). The iReAct study was an interdisciplinary research project which investigated the physiological, affective, and cognitive responses to HIIT and MICT at the individual level (Thiel et al., 2020). We used a two‐period sequential training intervention, where previously sedentary healthy participants were randomly assigned to 6 weeks of HIIT followed by 6 weeks of MICT exercise training or vice‐versa. Detailed information may be seen below and in our clinical trial protocol (Thiel et al., 2020). In the present manuscript, we analyzed the effects of the first training period only.

### 2.1 Recruitment and inclusion criteria

Participants were recruited primarily through the University of Tübingen and the University Hospital of Tübingen mailing list. Interested individuals were presented with detailed information regarding the study protocol and filled out the European Health Interview Survey‐Physical Activity Questionnaire (EHIS‐PAQ) (Finger et al., 2015) in order to assess their current physical activity levels. Participants that informed having less than 60 min per week of exercise during leisure time (including sports participation, aerobic activities, muscle strengthening) and no regular exercise for the past 6 months were eligible. Potential participants were then scheduled for a medical screening where they went through a standard anamnesis and blood draw. Participants that: presented a healthy status through the medical screening; did not have a body mass index (BMI) greater than 30 kg m^−2^ through their life course; and did not present signs of anemia (iron deficiency) in their blood results were eligible to start our diagnostics protocol. Males and females aged between 20 and 40 years old were recruited for the study. It should be noted that even though the phase of the menstrual cycle status within which testing occurred was not controlled in this study, this was not a major consideration in our design as there is compelling evidence showing that the phase of the menstrual cycle does not seem to have an effect on submaximal and maximal outcomes, as well as on microvascular measures (Mattu et al., 2020; Williams et al., 2020). Further inclusion criteria may be found elsewhere (Thiel et al., 2020).

### 2.2 Included participants

A total of 58 participants were assessed for eligibility, 49 of whom were included in the randomization process. Nine of them were excluded during medical diagnosis. Out of these nine excluded participants, seven were excluded for not meeting the inclusion criteria [iron deficiency anemia (*n* = 2), BMI above the predetermined upper limit (*n* = 1), under psychological treatment (*n* = 1), drug consumption (*n* = 1), and gastrointestinal issues (*n* = 2)], and two due to time management issues. During baseline measurements, five participants dropped out due to time management issues (*n* = 1), withdrawal during the acute exercise test due to discomfort with the exercise (*n* = 1), lack of willingness to continue participation (*n* = 1), a migraine episode (*n* = 1), and lung condition being discovered (*n* = 1). Therefore, 44 participants engaged in the exercise training intervention (HIIT *n* = 22 and MICT *n* = 22). One participant in each group dropped out during the exercise training intervention due to illness and not being able to complete the minimum adherence. A total of 42 participants (21 in each group) completed the study. Participants were informed of the experimental protocol and all associated risks prior to giving written informed consent. All procedures were conducted to conform with the Declaration of Helsinki and were approved by the Ethics Committee of the Medical Faculty of the University of Tübingen (882/2017BO1). An overview of participants’ characteristics is shown in Table 1.

**TABLE 1:** Overview of participants’ characteristics

	Responders, *N* = 31^a^	Non‐responders, *N* = 11^a^	*p*‐value
Sex			
Females	24 (77%)	6 (55%)	
Males	7 (23%)	5 (45%)	
Anthropometrics			
Age (yr)	26 ± 5	30 ± 6	0.068
BMI (kg/m²)	22.9 ± 2.2	25.7 ± 2.4	0.003
Height (cm)	170.5 ± 8.7	172.9 ± 9.7	0.483
Weight (kg)	66.6 ± 8.8	77.2 ± 12.5	0.011
Fitness			
Absolute ${\dot{V}O}_{2\max}$ (L·min^−1^)	2.24 ± 0.44	2.40 ± 0.39	0.036
Relative ${\dot{V}O}_{2\max}$ (ml·kg^−1^·min^−1^)	33.6 ± 4.8	31.5 ± 4.4	0.852
Blood markers			
Creatinine (mg/dL)	0.74 ± 0.16	0.74 ± 0.14	0.919
CRP (mg/dL)	0.18 ± 0.51	0.32 ± 0.46	0.100
Erythrocytes (Mio/µL)	4.66 ± 0.33	5.1 ± 0.5	0.007
Gamma‐GT (U/L)	16 ± 10	24 ± 21	0.500
Glucose (mg/dL)	82 ± 9	81 ± 5	0.863
GOT (U/L)	13.65 ± 4.24	15.64 ± 4.37	0.201
GPT (U/L)	18 ± 10	26 ± 15	0.257
Hematocrit (%)	39.9 ± 2.8	42.5 ± 4.8	0.126
Hemoglobin (g/dL)	13.77 ± 1.06	14.59 ± 1.75	0.213

Abbreviations: BMI, body mass index; ${\dot{V}O}_{2\max}$, maximal oxygen uptake; CRP, C‐reactive protein; Gamma‐GT, gamma‐glutamyl transferase; GOT, glutamic‐oxaloacetic transaminase; GPT, glutamic‐pyruvic transaminase.

^a^ Statistics presented: n (%); mean ± standard deviation.

### 2.3 Experimental design

**FIGURE 1:**
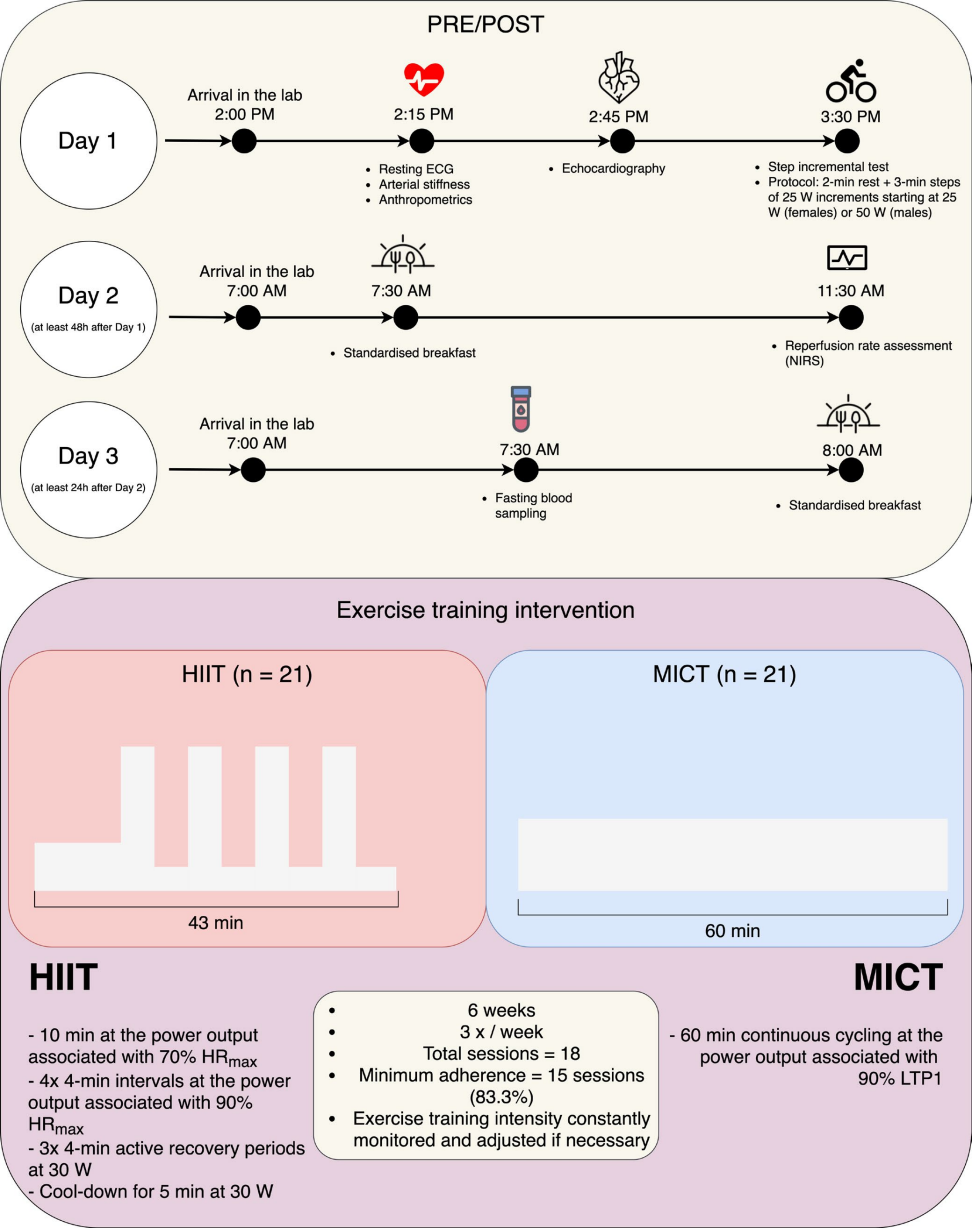
Overview of the experimental protocol design at pre‐training (PRE) and post‐training (POST). An overview of the exercise training prescription is displayed with the corresponding exercise times and intensities. Note: ECG, electrocardiogram; W, watts; NIRS, near‐infrared spectroscopy; HR_max_, maximal heart rate; LTP1, lactate turning point 1; HIIT, high‐intensity interval training; MICT, moderate‐intensity continuous training

In the present study, all physiological variables were assessed before (PRE) and after (POST) the 6 weeks of exercise training. An overview of the experimental design is displayed in Figure 1. PRE and POST measures were always divided into three separate testing days.

#### 2.3.1 Day 1

After arrival in the laboratory, participants’ measures of anthropometrics were taken, which included height, weight, and bioimpedance analysis (InBody770, InBody) for the estimation of body composition (i.e., body fat and muscle mass). Afterward, participants went to the s (ECG) room where they rested for 10 min in the supine position. Thereafter, the resting ECG was conducted for 2 min with participants still laying down (12‐channel PC ECG, custo med GmbH). After the ECG recording, participants remained quiet in the supine position for the assessment of arterial stiffness (BOSO ABI system 100, BOSO), as previously described (Diehm et al., 2009). Measures of the ankle‐brachial index (ABI), brachial‐ankle pulse wave velocity (baPWV), and carotid‐femoral pulse wave velocity (cfPWV) were derived (Diehm et al., 2009). Upon the completion of the ECG assessment, participants went to the echocardiography room, where a resting echocardiogram was performed (Philips IE 33, Philips) by a physician. Subsequently, participants undertook a step‐incremental test to volitional exhaustion on a cycle ergometer (Ergoselect 200, Ergoline GmbH) for the determination of ${\dot{V}O}_{2\max}$, peak power output, and the lactate thresholds [lactate turning point 1 (LTP1) and lactate turning point 2 (LTP2)]. Before starting the test, resting blood pressure and capillary blood lactate concentrations ([La^−^]) were measured. The test began with a 2‐min resting period on the bike, followed by 25‐W step increments every 3 min, starting at 50 W for males, and at 25 W for females, until task failure. [La^−^] was analyzed (Biosen S‐Line, EKF) by collecting capillary blood samples (20 µl) from the right earlobe during the last 20 s of each stage and right after volitional exhaustion. Blood pressure was taken again at 100 W and immediately after volitional exhaustion. Heart rate and ECG were constantly monitored throughout the test (12‐channel PC ECG, custo med GmbH). Breath‐by‐breath pulmonary gas exchange and ventilation were measured using a metabolic cart (MetaLyzer, CORTEX Biophysics). Calibration was performed before each test following the manufacturer's instructions.

#### 2.3.2 Day 2

Participants were instructed to come to the laboratory fasted (i.e., usually between 9 and 10 h). Upon arrival, a standardized breakfast was consumed (two cereal bars totalizing 160 kcal). Approximately three and a half hours after the breakfast, participants underwent a microvascular responsiveness assessment in the lower limb using near‐infrared spectroscopy (NIRS) (PortaMon, Artinis Medical Systems). After a 10‐min resting period on an examination table, the NIRS probe was placed on the muscle belly of the *tibialis anterior* muscle. The NIRS probe was secured with a black elastic strap to avoid movement and covered with a black vinyl sheet to minimize the intrusion of external light. A pneumatic cuff (Flexiport; Welch Allyn Inc.) was placed right below the knee (approximately 5 cm distal to the popliteal fossa) to induce blood flow occlusion when inflated. After the resting period, the cuff was instantaneously inflated to 260 mmHg for the whole duration of the occlusion time (5 min). Once the 5 min of occlusion were reached, the pressure of the cuff was instantaneously released, and the NIRS signal was recorded for 8 more minutes (McLay, Fontana, et al., 2016; McLay et al., 2016).

#### 2.3.3 Day 3

Participants were instructed to come to the laboratory fasted (i.e., usually between 9 and 10 h). Upon arrival, a fasting blood sampling was taken and the same standardized breakfast was consumed as on *Day 2*.

For baseline and follow‐up assessments, *Day 2* was only allowed to be performed at least 48 h after *Day 1* took place, whereas *Day 3* could be performed 24 h after *Day 2*. On *Day 1* in POST was only allowed to take place at least 48 h after the last training session.

### 2.4 Resting ECG and arterial stiffness

After the 10‐min resting period was completed, resting ECG was recorded for 2 min, and the resting heart rate was calculated as the average response over this duration. Arterial stiffness was measured as the brachial‐ankle index and the carotid‐femoral pulse wave velocity (Baulmann et al., 2010; Diehm et al., 2009).

### 2.5 Echocardiography

The echocardiography was performed by three routine echocardiographic physicians. All measurements were performed according to the standard recommendations and guidelines of the American Society of Echocardiography (ASE) and the European Society of Cardiology (ESC) (Lang et al., 2005; Nagueh et al., 2009; Rudski et al., 2010) to ensure high quality and valid data collection. In order to adjust the echocardiographic parameters to the cardiac cycle, a device‐integrated ECG was used. The collected echocardiographic data were digitally stored and subsequently analyzed by the respective physician using the internal software. The storage of the collected data (including images and loops over five heartbeats) was achieved by image transfer to the department's internal software. The following measures were derived from the resting echocardiography:

#### 2.5.1 Left ventricle and left atrium morphology

Left ventricular end‐diastolic diameter (LVEDD), left ventricular mass (LV‐Mass), LV‐Mass index (LVMI), absolute heart volume, left atrium M‐Mode (LA M‐Mode), and left atrium size by planimetry (LA planimetric).

#### 2.5.2 Right ventricle and right atrium morphology

Right ventricular end‐diastolic diameter (RVEDD), and right atrium size by planimetry (RA planimetric).

#### 2.5.3 Left ventricular systolic function

Fractional shortening (FS), ejection fraction by the Simpson method (EF Simpson), mean of mitral annular plane systolic excursion (MAPSE mean) – calculated as the mean between MAPSE septal and lateral, and mean of left ventricular excursion velocity (s’ LV mean) – calculated as the mean between s’ LV septal and lateral.

#### 2.5.4 Left ventricular diastolic function

The ratio between the E‐wave (i.e., mitral inflow velocity) and the A‐wave (i.e., atrial inflow velocity) (E/A), ratio between the mean of diastolic mitral velocity (E’ mean) – calculated as the mean between the E’ septal and lateral – and the mean of diastolic atrial velocity (A’ mean) – calculated as the mean between the A’ septal and lateral (E’ mean/A’ mean), and the E/E’ ratio.

#### 2.5.5 Right ventricular systolic function

Tricuspid annular plane systolic excursion (TAPSE) and right ventricular excursion velocity (s’ RV).

### 2.6 Step incremental test to exhaustion measures

Breath‐by‐breath oxygen uptake (${\dot{V}O}_{2}$) data were edited as follows: Breaths (data points) that were two standard deviations (95% of prediction interval) away from the local mean were considered outliers and then removed. Thereafter, the data were interpolated on a second‐by‐second basis and averaged into 30‐s bins for ${\dot{V}O}_{2\max}$ analysis (Mattioni Maturana et al., 2018).

#### 2.6.1 Maximal values

${\dot{V}O}_{2\max}$ was considered the highest 30‐s ${\dot{V}O}_{2}$ average. ${\dot{V}O}_{2\max}$ attainment was confirmed if at least two of the following three criteria were met, as per the American College of Sports Medicine guidelines (American College of Sports Medicine et al., 2018): (i) maximal heart rate within 10 beats per minute (bpm) of the maximal predicted value (220 – age); (ii) a respiratory exchange ratio (RER) higher than 1.10; or (iii) a maximal [La^−^] of 8 mmol·L^−1^. It has been previously proposed a verification ride to confirm the attainment of ${\dot{V}O}_{2\max}$ (Rossiter et al., 2006). However, several investigations have demonstrated that this procedure does not add confidence to the evaluation beyond what the incremental test to task failure offers (Iannetta et al., 2020a; Murias et al., 2018; Wagner et al., 2021). Peak power output (PO_peak_) was considered as the value achieved at the moment of exhaustion and the maximal heart rate (HR_max_) was considered as the highest value throughout the test. To calculate the exact PO_peak_ achieved in case the stage was not completed, a linearization of the power output was performed, such that the end power output of each stage would correspond to the stage power output.

#### 2.6.2 LTP1 and LTP2

Lactate thresholds were analyzed using a segmented regression model at which two breakpoints were estimated from the [La^−^]‐power output relationship. LTP1 was determined as the first rise in [La^−^] above baseline levels (first breakpoint) (Binder et al., 2008; Hofmann & Tschakert, 2017). LTP2 was determined as the second abrupt increase in [La^−^] (second breakpoint) (Binder et al., 2008; Hofmann & Tschakert, 2017). All of these measures were analyzed as a function of power output, and then their corresponding ${\dot{V}O}_{2}$ values were analyzed from the ${\dot{V}O}_{2‐}$ power output relationship. In order to minimize the effect of the ${\dot{V}O}_{2}$ mean response time (i.e., a delay in the ${\dot{V}O}_{2}$ response to the increase in exercise intensity causing a mismatch between the increase in ${\dot{V}O}_{2}$ and power output), we used the ${\dot{V}O}_{2}$ average of the last 30 s of each step to calculate the slope and intercept of the ${\dot{V}O}_{2‐}$ power output relationship.

#### 2.6.3 Efficiency slopes

The oxygen uptake efficiency slope (OUES), which is an index of the cardiopulmonary functional reserve, was determined as the relationship between ${\dot{V}O}_{2}$ (in ml·min^−1^) and $\dot{V}E$ (Baba et al., 1996; Hollenberg & Tager, 2000; Onofre et al., 2017). OUES is analyzed as the slope of the following linear equation:

$$\dot{V}O_{2}=a\times \log_{10}\dot{V}E+b.$$

The ${\dot{V}O}_{2‐}$ power output $\left(\Delta {\dot{V}O}_{2}/\Delta \text{PO}\right)$ slope, which is a surrogate of the efficiency of the aerobic metabolism to provide energy, was determined by linear regression using the least square method (Hansen et al., 1988; Prieur et al., 2005). The heart rate‐${\dot{V}O}_{2}$ $\left(\Delta \text{HR}/\Delta {\dot{V}O}_{2}\right)$ slope, which is an index of stroke volume and peripheral oxygen extraction, was also determined by linear regression using the least square method (Fairbarn et al., 1994; Neder et al., 2001; Spiro et al., 1974).

### 2.7 Microvascular responsiveness

Microvascular responsiveness was assessed as previously described (McLay, Nederveen, et al., 2016). Shortly, the baseline oxygen saturation (StO_2_) was calculated as the average of the last 2 min of the baseline period prior to ischemia. The StO_2_ reperfusion rate (slope 2) was calculated as the slope of the StO_2_ signal during the first 10 s immediately after cuff release. The StO_2_ area under the curve was calculated as the total area under the curve of the StO_2_ signal during the reperfusion phase using the trapezoid method. For the area under the curve calculation, only values above the baseline during the first 4 min of the reperfusion period were considered.

### 2.8 Fasting blood parameters

Fasting blood parameters were analyzed via spectrophotometric detection of hexokinase activity. Insulin resistance was defined as the insulin resistance index (homeostasis model assessment, HOMA‐IR), which was calculated according to the formula:

$$\mathrm{HOMA}‐\mathrm{IR}=\frac{\mathrm{fasting}\;\mathrm{insulin}\;\times \mathrm{fasting}\;\mathrm{glucose}}{22.5},$$

where fasting insulin is in mU/L and fasting glucose is in mmol/L.

### 2.9 Exercise training intervention

The HIIT and MICT prescriptions were designed aiming to match both interventions by energy expenditure. A literature search was performed in order to gather information on previous studies matching energy expenditure for HIIT and MICT (Helgerud et al., 2007; Mitranun et al., 2014; Molmen‐Hansen et al., 2012; Nie et al., 2018; Ramos et al., 2016; Rognmo et al., 2004; Tjonna et al., 2008; Winn et al., 2018). After careful consideration, we prescribed the following exercise training programs (Figure 1 displays an overview of the prescriptions):

#### 2.9.1 HIIT

The HIIT group performed 10 min of warm‐up at the power output corresponding to 70% of their HR_max_, followed by four 4‐min intervals at the power output corresponding to 90% of their HR_max_. Each high‐intensity interval was interspersed with a 4‐min active recovery at 30 W. After the last high‐intensity interval, a 5‐min cool‐down at 30 W was performed, totalizing 43 min of exercise. The power output at each percentage of HR_max_ was derived from the ∆HR/∆PO relationship during the step incremental test performed on *Day 1*. To account for the delay in the heart rate response to the increase in work rate in each step, the average of the last 30 s of each step was taken, and then plotted against power output, deriving the linear model used for the calculation. The exercise intensity at 90% of HR_max_ was also chosen given the fact that such intensity is most likely to place individuals within the severe‐intensity domain (Iannetta, Inglis, et al., 2020).

#### 2.9.2 MICT

The MICT group performed 60 min of continuous cycling at the power output corresponding to 90% of LTP1. Such exercise intensity was prescribed for participants to cycle within the moderate‐intensity domain (Binder et al., 2008; Hofmann & Tschakert, 2017).

#### 2.9.3 Training monitoring

All exercise training sessions were performed on a cycle ergometer (ec5000, custo med GmbH) and participants’ heart rate and ECG were constantly monitored (3‐channel ECG, custo med GmbH). After every training session, the exercise training data (i.e., second‐by‐second power output, cadence, and heart rate) were exported and stored for subsequent processing.

#### 2.9.4 Minimum adherence

In order for participants to be included in the final analyses, a minimum of 15 out of the 18 prescribed exercise sessions had to be completed (minimum adherence =83.3%). If participants did not complete the minimum required number of sessions, they were considered as dropouts (*n* = 2).

### 2.10 Training analyses

From the power output and heart rate data collected in each training session, the following was calculated:

#### 2.10.1 Power output

Measures of power output were derived as follows: percentage of peak power output (%PO_peak_), total work (power output × time), relative total work (total work normalized to body weight), total kcal (total work / 4.184), and relative total kcal (total kcal normalized to body weight). All the above measures were analyzed as a mean across the whole sessions and adjusted values for the HIIT were also calculated (i.e., averages only considering the power output during the high‐intensity intervals).

#### 2.10.2 Heart rate

As aforementioned, prior to calculating the heart rate associated with each training session, the data were cleaned, interpolated on a second‐by‐second basis, and bin‐averaged into 5‐s bins. Thereafter, the mean heart rate associated with every training session was derived (also expressed as a percentage of HR_max_ and heart rate reserve), as well as the individual training impulse (iTRIMP) (Manzi et al., 2009; Sanders et al., 2017). Briefly, the iTRIMP method uses the individual's heart rate‐blood lactate relationship from an incremental test to exhaustion to calculate an exponential factor for each individual. This weighting factor is then used to calculate the training impulse, which is an integration of exercise training duration, average heart rate of the training session, and the individual exponential factor.

### 2.11 Responders’ classification

**FIGURE 2:**
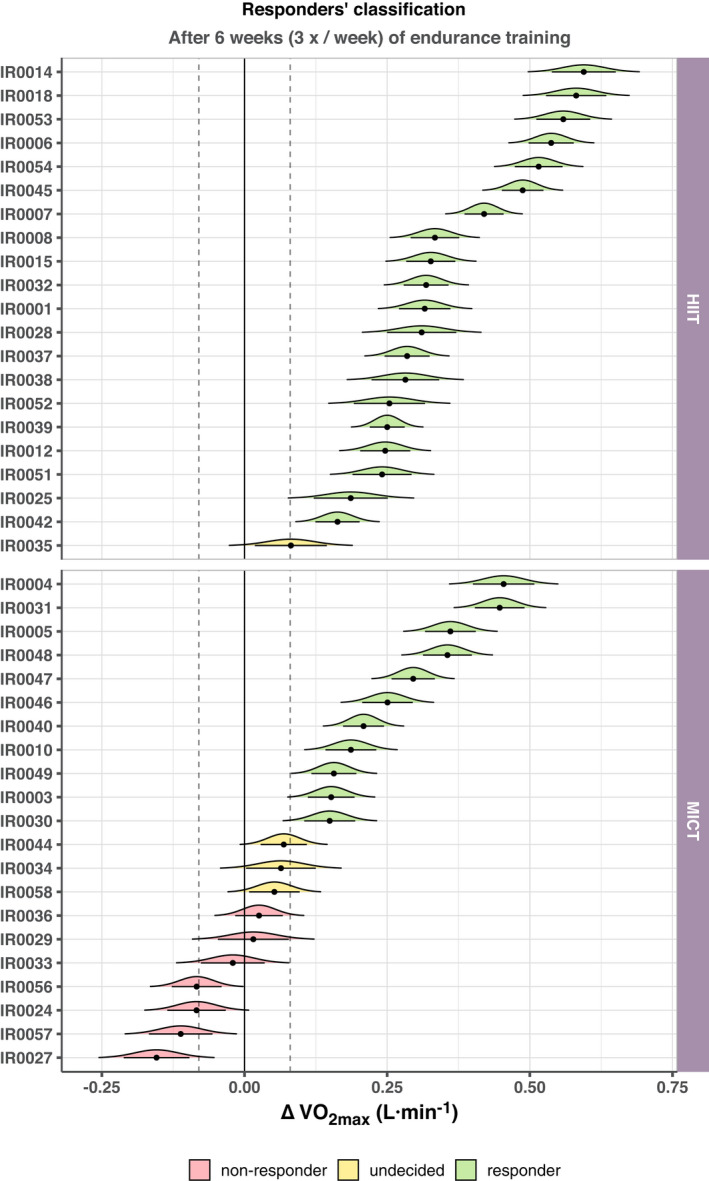
Overview of responders’ classification using the highest density interval (HDI) and region of practical equivalence (ROPE) method. The graph displays the $\Delta {\dot{V}O}_{2\max}$ (delta of maximal oxygen uptake) of each participant. The black dots show the $\Delta {\dot{V}O}_{2\max}$, curves are the normal distribution curves derived for each individual given the measurement error around the $\Delta {\dot{V}O}_{2\max}$ value. The horizontal black lines are the 89% HDI derived from each curve, and vertical dashed lines around zero are the calculated ROPE (i.e., −80 to 80 ml·min^−1^). Please, refer to Table 3 for an overview of the levels of significance from the ROPE + HDI decision‐making method applied to responders’ analysis

The responders’ classification was performed using the ROPE + HDI decision rule (Kruschke, 2018; Maturana et al.,). This Bayesian decision‐making method uses the region of practical equivalence (ROPE) in combination with the highest density interval (HDI) as the basis for accepting or rejecting the null hypothesis. The HDI summarizes the most credible values of a parameter (similar to the confidence interval in frequentist statistics), while the ROPE provides a range of values around the null value. Therefore, unlike null‐hypothesis testing where values are tested against zero, the ROPE + HDI method calculates the percentage of HDI that is within the ROPE. Based on this percentage, there are different levels of significance, which we then apply to responders’ classification (see Figure 2 for an overview and (Kruschke, 2018) for an introduction to the topic). The following steps were taken:

#### 2.11.1 Step 1 – calculate the $\Delta {\dot{V}O}_{2\max}$ and its associated measurement of error

The $\Delta {\dot{V}O}_{2\max}$ was analyzed as the raw difference (∆ = POST – PRE) from the absolute ${\dot{V}O}_{2\max}$ response (i.e., L·min^−1^). We additionally considered the technical error of measurement around the $\Delta {\dot{V}O}_{2\max}$ as the coefficient of variation associated with ${\dot{V}O}_{2\max}$ measures (i.e., 5.6%), which also accounts for the random variation of true changes (Hecksteden et al., 2015, 2018). This works as an external reliability measure, which uses previously published coefficient of variation associated with ${\dot{V}O}_{2\max}$ measures when repeated testing is not available. Therefore, the coefficient of variation was calculated as 5.6% of the baseline ${\dot{V}O}_{2\max}$ for each individual and a range around the $\Delta {\dot{V}O}_{2\max}$ was obtained:

$$\mathrm{measurement}\;\mathrm{error}=\frac{\mathrm{baseline}\;\dot{V}O_{2\max}\times 5.6\%}{2}$$

$$\mathrm{individual}\;\mathrm{range}=\;\left[\Delta \dot{V}O_{2\max}‐\mathrm{measurement}\;\mathrm{error};\Delta \dot{V}O_{2\max}+\mathrm{measurement}\;\mathrm{error}\right]$$

Once the individual range was obtained, we then calculated the rough estimate of the standard deviation around the individual $\Delta {\dot{V}O}_{2\max}$:

$$\Delta_{\mathrm{sd}}=\frac{\mathrm{individual}\;\mathrm{range}}{4},$$

where ∆_sd_ is the individual rough estimate for the standard deviation around the $\Delta {\dot{V}O}_{2\max}$.

#### 2.11.2 Step 2 – derive a normal distribution for each individual

Thereafter, once we have the mean ($\Delta {\dot{V}O}_{2\max}$) and standard deviation (∆_sd_) from each individual, we derived a normal distribution for each one of them (calculated from simulated 100 measures that spanned across all the possible ranges):

$$f\left(x\right)=\frac{1}{\sigma \sqrt{2\pi }}e^{‐\frac{1}{2}{\left(\frac{x‐\mu }{\sigma }\right)}^{2}},$$

where σ is the $\Delta {\dot{V}O}_{2\max}$ and µ is the ∆_sd_.

#### 2.11.3 Step 3 ‐ calculate the HDI and ROPE

The HDI was retrieved from each individual normal distribution, calculated as 89% of the credible interval (Kruschke, 2015, 2018). The ROPE was defined as the clinically relevant difference, calculated as the smallest worthwhile difference (Hecksteden et al., 2015; Hopkins et al., 1999). In practical terms, the ROPE was set as 20% of the baseline ${\dot{V}O}_{2\max}$ standard deviation in both directions (i.e., ROPE = −80 to 80 ml·min^−1^. Full ROPE range = 160 ml·min^−1^).

#### 2.11.4 Step 4 – responder classification

Each participant was then classified according to the HDI percentage within the ROPE. In the Bayesian framework, the percentage within the ROPE has different levels of significance (Makowski et al., 2019a). We then applied these levels labels to the responders’ classification. Participants that presented a negative $\Delta {\dot{V}O}_{2\max}$ were all considered as non‐responders. In order to assess whether the regression to the mean phenomenon was present in the ${\dot{V}O}_{2\max}$ results, we performed an analysis of covariance (ANCOVA), as previously suggested (Barnett, 2004).

### 2.12 Statistical analyses

Results are presented as mean ± standard deviation unless otherwise stated.

A linear mixed model (estimated using restricted maximum likelihood) was performed on the repeated measures of each parameter using the *responders’ classification* and *training phase* as main effects. Therefore, we compared responders and non‐responders in PRE and POST. First, we performed within‐subject comparisons (i.e., repeated measures PRE vs. POST) and then between‐subject comparisons (i.e., responders vs. non‐responders). The model included the participants as random effects. For all the post hoc analyses, we computed estimate marginal means applying the Bonferroni method as multiple comparisons’ correction. Each linear mixed model was tested for multicollinearity of model terms by calculating the variance inflation factor (VIF) (James et al., 2013), outliers detection using the Cook's distance method (Cook, 1977), and normality of residuals.

All data analyses, editing, and visualizations were performed in R version 4.0.2 (R Core Team 2020) with the packages *tidyverse* (Wickham et al., 2019), *bayestestR* (Makowski et al., 2019b *)*, and *lme4* *(*Bates et al., 2015).

## 3 RESULTS

### 3.1 Exercise training intervention

The relative total work (mean across sessions) associated with responders (3.27 ± 0.58 kJ/kg) and non‐responders (3.23 ± 0.75 kJ/kg) was not statistically different (*p* = 0.87; Cohen's *d* = 0.06, 95% confidence interval [−0.63; 0.75]). Table 2 displays an overview of the mean values for % HR_max_, % PO_peak_, and iTRIMP of each week, as well as the mean relative total work across the 6 weeks of training.

**TABLE 2:** Exercise training data from the 6 weeks of training in HIIT and MICT. The percentage of maximal heart rate and peak output values are the mean across the sessions in each week, and the iTRIMP and relative total work values are the sum of the sessions in each week

		Week 1	Week 2	Week 3	Week 4	Week 5	Week 6
HIIT	*%HR_max_*	75.7 ± 3.5%	75.7 ± 3.2%	75.1 ± 3.8%	75.1 ± 3.7%	74.9 ± 4.2%	73.6 ± 3.7%
	*%HR_max_ adjusted*	88.9 ± 3.7%	89.5 ± 3.2%	88.8 ± 3.4%	88.8 ± 3.2%	89.5 ± 3.0%	88.4 ± 2.8%
	*%PO_peak_*	47.9 ± 4.2%	49.3 ± 3.6%	50.3 ± 3.7%	50.9 ± 3.8%	51.7 ± 3.6%	52.2 ± 3.6%
	*%PO_peak_ adjusted*	79.4 ± 5.8%	82.9 ± 4.3%	85.5 ± 4.6%	87.1 ± 5.0%	89.2 ± 4.6%	90.6 ± 4.9%
	*iTRIMP*	13824 ± 6549 A.U.	13400 ± 6992 A.U.	14053 ± 6257 A.U.	13362 ± 5741 A.U.	13631 ± 7258 A.U.	8352 ± 5450 A.U.
	*Relative total work*	8.9 ± 1.4 kJ/kg	9.2 ± 1.4 kJ/kg	9.4 ± 1.4 kJ/kg	9.5 ± 1.4 kJ/kg	9.6 ± 1.5 kJ/kg	6.7 ± 2.8 kJ/kg
MICT	*%HR_max_*	68.3 ± 6.4%	68.8 ± 6.5%	68.4 ± 6.0%	68.2 ± 6.4%	69.6 ± 6.0%	68.1 ± 6.8%
	*%PO_peak_*	39.4 ± 6.3%	40.2 ± 6.5%	41.2 ± 7.1%	42.4 ± 6.1%	43.7 ± 5.6%	43.4 ± 5.4%
	*iTRIMP*	7428 ± 4203 A.U.	7898 ± 5259 A.U.	6964 ± 3749 A.U.	7070 ± 4370 A.U.	8057 ± 5478 A.U.	4361 ± 4409 A.U.
	*Relative total work*	9.6 ± 2.1 kJ/kg	9.9 ± 2.3 kJ/kg	10.1 ± 2.4 kJ/kg	10.3 ± 2.1 kJ/kg	10.7 ± 2.1 kJ/kg	6.2 ± 2.8 kJ/kg

Note

%HR_max_ adjusted and %PO_peak_ adjusted are the percentage of maximal heart rate and percentage of peak output considering only the high‐intensity intervals.

### 3.2 Responders’ classification

All the participants achieved the minimum criteria for $\Delta {\dot{V}O}_{2\max}$ attainment in both PRE (HR_max_ = 190 ± 12 bpm [7 ± 6 bpm within the predicted HR_max_], maximal RER = 1.26 ± 0.08, and maximal [La^−^] = 9.4 ± 1.8 mmol·L^−1^) and POST (HR_max_ = 190 ± 11 bpm [8 ± 6 bpm within the predicted HR_max_], maximal RER = 1.27 ± 0.05, and maximal [La^−^] = 10.4 ± 1.9 mmol·L^−1^). In general, the exercise training intervention increased ${\dot{V}O}_{2\max}$ significantly with a large effect size (+240 ± 196 ml·min^−1^ or +12 ± 10%; *p* < 0.001, *d* = 1.23 95% CI: [0.83; 1.64]). Additionally, the HIIT group showed a greater increase in ${\dot{V}O}_{2\max}$ than the MICT group (mean difference ±standard error: 214 ± 36 ml·min^−1^, *p* < 0.001). The summary of the individual responders’ classification is shown in Figure 2. The “non‐responders” and “undecideds” were aggregated into “non‐responders” for subsequent statistical analyses. Altogether, there were 31 “responders” and 11 “non‐responders.” Table 3 displays an overview of the decision‐making process based on the ROPE + HDI approach described in the methods. These results were confirmed by an ANCOVA, also showing a significant increase in ${\dot{V}O}_{2\max}$ adjusted by baseline values (ß = 1.01, 95% CI = [0.91; 1.12], *p* < 0.001), and between responders and non‐responders (ß = 0.35, 95% CI = [0.25; 0.44], *p* < 0.001).

**TABLE 3:** Region of practical equivalence (ROPE) and highest density interval (HDI) decision‐making applied to responder's analysis

HDI % in ROPE	ROPE + HDI levels of significance	Responders’ levels of significance
>99%	negligible (accept null hypothesis)	non‐responder
>97.5%	probably negligible	probably non‐responder
≤97.5% & ≥2.5%	undecided significance	undecided
<2%	probably significant	probably responder
<1%	significant (reject null hypothesis)	responder

### 3.3 Adaptations in responders and non‐responders

**FIGURE 3:**
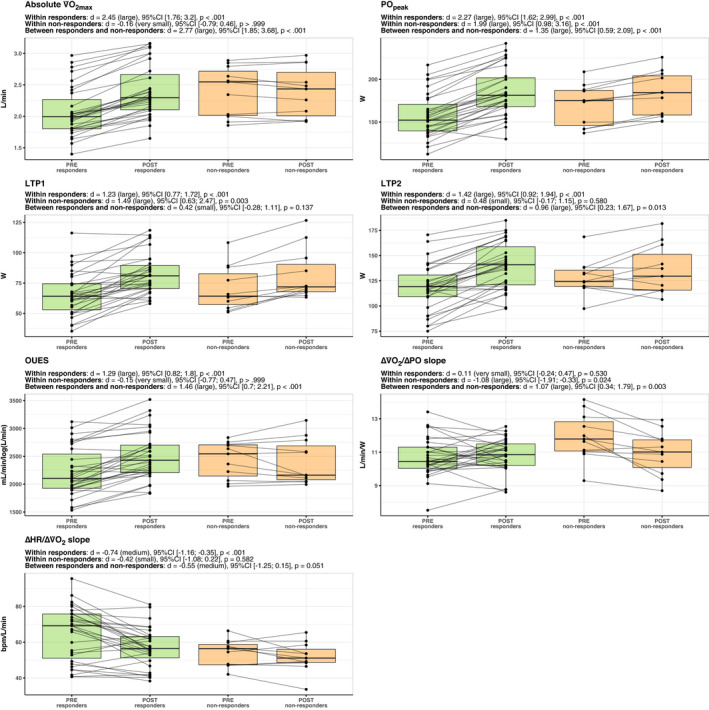
Within‐ and between groups differences between responders and non‐responders in the fitness‐related parameters. The within‐ and between‐subject comparisons are displayed as effect sizes (Cohen's d), 95% confidence intervals, and adjusted p‐values. The black dots display the individual values, which are connected from PRE to POST through continuous lines. Note: ${\dot{V}O}_{2\max}$, maximal oxygen uptake; POpeak, peak power output; LTP1, lactate turning point 1; LTP2, lactate turning point 2; OUES, oxygen uptake efficiency slope; $\Delta {\dot{V}O}_{2}/\Delta \text{PO}$ slope, slope of the oxygen uptake‐power output relationship during the incremental test to exhaustion; $\Delta \text{HR}/\Delta {\dot{V}O}_{2}$ slope, slope of the heart rate‐oxygen uptake relationship during the incremental test to exhaustion

**FIGURE 4:**
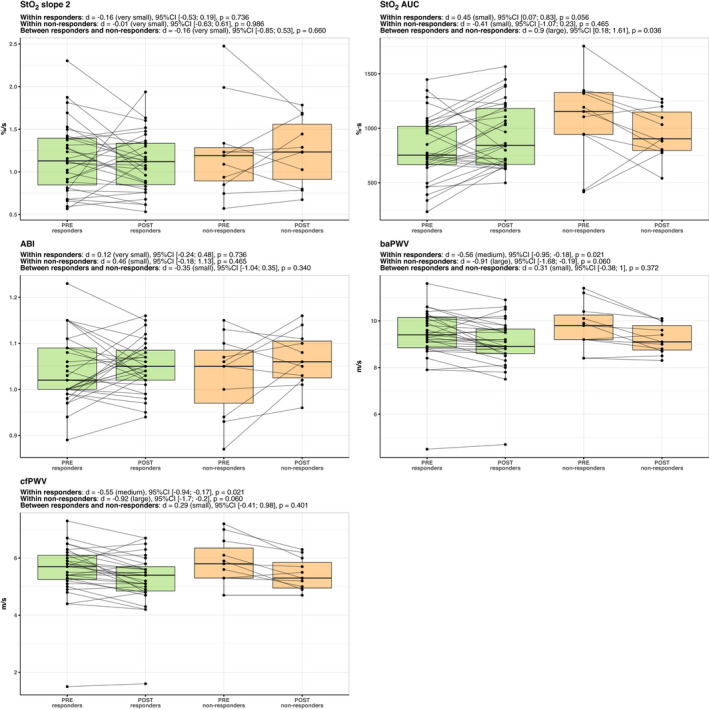
Within‐ and between groups differences between responders and non‐responders in the vascular‐ and microvascular‐related parameters. The within‐ and between‐subject comparisons are displayed as effect sizes (Cohen's d), 95% confidence intervals, and adjusted p‐values. The black dots display the individual values, which are connected from PRE to POST through continuous lines. Note: StO_2_ slope 2, NIRS‐derived oxygen saturation slope 2; StO_2_ AUC, NIRS‐derived oxygen saturation area under the curve; ABI, ankle‐brachial index; baPWV, brachial‐ankle pulse wave velocity; cfPWV, carotid‐femoral pulse wave velocity

**FIGURE 5:**
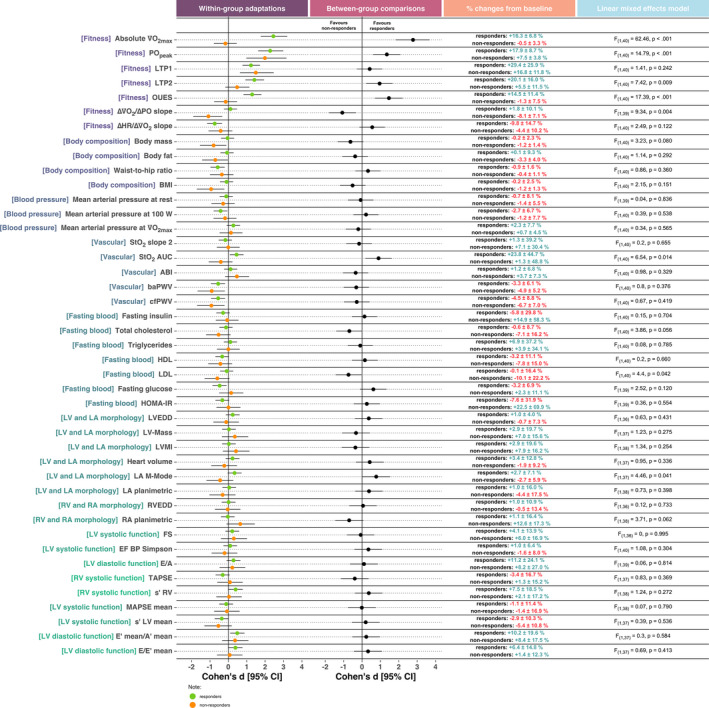
Overview of the effect sizes (Cohen's d) and 95% confidence intervals on each one of the examined variables. The first column shows within‐group effect sizes, the second column shows between‐groups effect sizes, the third column displays the percent changes from baseline in responders and non‐responders, and the fourth column displays the results from the interaction effects (responders’ classification x training phase) from the linear mixed effect model

Figures 3 and 4 display an overview of the within‐ and between‐responder's classifications adaptations in the fitness and vascular parameters showing the individual changes. All the other parameters are shown in Figures [Link], [Link], [Link], [Link], [Link], [Link], [Link], [Link]. Figure 5 shows an overview of the effect sizes.

Fitness: The linear mixed model (responder's classification x training phase) revealed a significant interaction effect in increasing the absolute ${\dot{V}O}_{2\max}$ (F_(1, 40)_ = 62.5, *p* < 0.001), PO_peak_ (F_(1, 40)_ = 14.8, *p* < 0.001), LTP2 (F_(1, 40)_ = 7.42, *p* < 0.01), and OUES (F_(1, 40)_ = 17.4, *p* < 0.001), and reducing the $\Delta {\dot{V}O}_{2}/\Delta \text{PO}$ slope (F_(1, 40)_ = 9.34, *p* = 0.004). Additionally, there was a main effect for training phase (pre vs. post‐training) in increasing LTP1 (F_(1, 40)_ = 42.3, *p* < 0.001) and decreasing the $\Delta \text{HR}/\Delta {\dot{V}O}_{2}$ slope (F_(1, 40)_ = 9.42, *p* = 0.004). Within‐group comparisons indicated that responders presented a significant difference pre‐ to post‐training in ${\dot{V}O}_{2\max}$ (*p* < 0.001), PO_peak_ (*p* < 0.001), LTP1 (*p* < 0.001), LTP2 (*p* < 0.001), OUES (*p* < 0.001), and $\Delta \text{HR}/\Delta {\dot{V}O}_{2}$ slope (*p* < 0.001). Non‐responders presented a significant difference pre‐ to post‐training in PO_peak_ (*p* < 0.001), LTP1 (*p* = 0.003), and $\Delta {\dot{V}O}_{2}/\Delta \text{PO}$ slope (*p* = 0.024). Post hoc analyses on the ∆ (i.e., change between PRE and POST) revealed significant differences between responders and non‐responders favoring a greater increase in responders in absolute ${\dot{V}O}_{2\max}$ (*d* = 2.77, 95% CI:[1.85; 3.68], *p* < 0.001), PO_peak_ (*d* = 1.35, 95% CI:[0.59; 2.09], *p* < 0.001), LTP2 (*d* = 0.96, 95% CI:[0.23; 1.67], *p* = 0.013), and OUES (*d* = 2.77, 95% CI:[1.85; 3.68], *p* < 0.001). The non‐responders group presented a significantly greater reduction than responders in the $\Delta {\dot{V}O}_{2}/\Delta \text{PO}$ slope parameter (*d* = 1.07, 95% CI:[0.34; 1.79], *p* = 0.003).

#### 3.3.1 Body composition

No interaction effects were observed in the linear mixed model in body composition measures. A main effect for responder's classification was observed in reducing body mass (F_(1, 40)_ = 8.53, *p* = 0.006), BMI (F_(1, 40)_ = 11.3, *p* = 0.002), and waist‐to‐hip ratio (F_(1, 40)_ = 11.9, *p* = 0.001). Additionally, the main effect for training phase (pre vs. post‐training) was observed in reducing body mass (F_(1, 40)_ = 4.21, *p* = 0.047), BMI (F_(1, 40)_ = 4.31, *p* = 0.044), and in waist‐to‐hip ratio (F_(1, 40)_ = 6.57, *p* = 0.014).

#### 3.3.2 Blood pressure

No interaction or main effects were observed in the linear mixed model in body pressure measures.

#### 3.3.3 Vascular function tests

The linear mixed model (responder's classification × training phase) revealed a significant interaction effect in increasing StO_2_ AUC (F_(1, 40)_ = 6.54, *p* = 0.014). Post hoc analyses revealed a large effect size favoring responders in increasing StO_2_ AUC (*d* = 0.90, 95% CI:[0.18; 1.61], *p* = 0.036). Additionally, the main effect for training phase (pre vs. post‐training) was observed in reducing baPWV (F_(1, 40)_ = 16.7, *p* < 0.001) and cfPWV (F_(1, 40)_ = 16.4, *p* < 0.001), reflecting a decrease in arterial stiffness after training. Within group analyses indicated a significant reduction in baPWV (*p* = 0.021) and cfPWV (*p* = 0.021) from pre‐ to post‐training. No significant differences were observed in non‐responders.

#### 3.3.4 Fasting blood outcomes

There was a significant interaction effect (responder's classification × training phase) for reducing LDL (F_(1, 40)_ = 4.40, *p* = 0.042). Additionally, there was a main effect for responder's classification favoring a decrease in fasting insulin (F_(1, 40)_ = 7.14, *p* = 0.01), triglycerides (F_(1, 40)_ = 9.76, *p* = 0.003), and insulin resistance (F_(1, 40)_ = 7.39, *p* = 0.01). The main effect for training phase (pre vs. post‐training) was observed favoring a decrease in total cholesterol (F_(1, 40)_ = 6.4, *p* = 0.015) and HDL (F_(1, 40)_ = 5.23, *p* = 0.028). Although not significant, between group analyses indicated that non‐responders presented a greater reduction than responders in LDL (*d* = 0.74, 95% CI: [0.03; 1.44], *p* = 0.115) and total cholesterol (*d* = 0.69, 95% CI: [−0.02; 1.39], *p* = 0.167).

#### 3.3.5 Cardiac evaluation

The mixed linear model revealed an interaction effect in increasing the LA M‐Mode – a marker of left atrium morphology (F_(1, 37)_ = 4.46, *p* = 0.041). Additionally, there was a main effect for training phase (pre vs. post‐training) in decreasing s’ LV mean – a marker of left‐ventricular systolic function (F_(1, 37)_ = 6.14, *p* = 0.018) and in increasing E’ mean/A’ mean – a marker of left‐ventricular diastolic function (F_(1, 37)_ = 4.25, *p* = 0.046). Post hoc analyses revealed a trend (*p* = 0.057) and a medium effect size (*d* = 0.77, 95% CI:[0.00; 1.53]) for greater increase in LA M‐Mode in the responders group compared to non‐responders.

## 4 DISCUSSION

To the best of our knowledge, this was the first study investigating whether the differences in ${\dot{V}O}_{2\max}$ responses to a short‐term exercise training intervention between responders and non‐responders would be accompanied by differences in body composition, vascular, metabolic, and cardiac adaptations. The main findings of the current study were that:

- In the fitness parameters, responders had a greater increase than non‐responders in PO_peak_, LTP2, and OUES, whereas non‐responders had a greater decrease than responders in the $\Delta {\dot{V}O}_{2}/\Delta \text{PO}$ slope—which is a marker of cycling efficiency. Additionally, non‐responders also presented a significant increase in PO_peak_ and LTP1 from pre‐ to post‐training, despite the lack of increase in ${\dot{V}O}_{2\max}$.
- The exercise training showed positive microvascular effects in responders, with a significant increase in StO_2_ AUC after training.
- No statistical differences were observed between responders and non‐responders in body composition, blood pressure, fasting blood parameters as well as resting cardiac adaptations.

### 4.1 Fitness

Alongside greater adaptations in ${\dot{V}O}_{2\max}$, responders also presented a greater increase in PO_peak_, LTP2, and OUES (i.e., a marker of respiratory efficiency). Interestingly, non‐responders showed a greater reduction in the $\Delta {\dot{V}O}_{2}/\Delta \text{PO}$ slope, a measure of cycling efficiency. In this context, it has already been shown that 5 weeks of moderate‐intensity endurance training significantly reduced the $\Delta {\dot{V}O}_{2}/\Delta \text{PO}$ slope, specially above the lactate threshold 1, and increased PO_peak_ even without significant increases in ${\dot{V}O}_{2\max}$ (Majerczak et al., 2012). Our results agree with these findings, as out of the 11 non‐responders, 10 were from the MICT group. Such enhancement could have been due to a lower ATP turnover at the same absolute power output during the incremental exercise—possibly an effect of the downregulation of the sarcoendoplasmic calcium ATPase pumps induced by the moderate‐intensity exercise (Walsh et al., 2006). These findings highlight the greater efficacy of MICT in improving cycling mechanical efficiency and its independency from the mechanisms responsible for the increase in ${\dot{V}O}_{2\max}$. Nevertheless, it is important to highlight that even participants who were considered “non‐responders” based on their ${\dot{V}O}_{2\max}$ response, showed other positive adaptations to exercise training (i.e., PO_peak_, LTP1, and $\Delta {\dot{V}O}_{2}/\Delta \text{PO}$ slope). Ultimately, the successful outcome of an exercise training intervention is going to depend on the target clinical endpoint in combination with the characteristics of the individual.

### 4.2 Vascular function

Both responders and non‐responders demonstrated some level of reduction in arterial stiffness as indicated by medium and large effect sizes, respectively. It must be acknowledged that these results are somewhat limited due to the lack of statistical significance in this outcome. However, it can be observed through our individual data that positive adaptations occurred consistently in both groups, despite the short‐term training intervention of 6 weeks (3× / week). The mechanisms underlying the beneficial effects of exercise on arterial stiffness are rather complex. Overall, regular exercise results in repeated exposure to periods of increased blood flow and consequently higher vascular wall shear stress. Intermittent increases in wall shear stress stimulate the endothelial cells to produce NO, which plays a crucial role in overall vascular health by decreasing inflammation and inhibiting the expression of vascular proteins associated with vascular stiffness development (Steppan et al., 2014).

At the microvascular level, our results showed that responders and non‐responders presented differences in leg skeletal muscle microvascular responsiveness following exercise training, which are in agreement with previous investigations (Soares et al., 2018, 2019). As further discussed elsewhere (Soares et al., 2019), NIRS‐derived reperfusion rate is mainly driven by the capacity of the downstream vasculature to dilate and increase capillary recruitment in response to ischemia. The vascular effects of ischemia would increase the capacity of the downstream microvasculature to accommodate more oxygenated blood originated from the upstream conduit arteries upon cuff release, thereby reflecting in a faster (StO_2_ slope 2) and/or prolonged overshoot of the skeletal muscle StO_2_ signal during reperfusion (StO_2_ AUC) (Rosenberry & Nelson, 2020). Exercise training has been associated with vascular adaptations such as the enlargement of conduit arteries, increased expression of genes encoding endothelial proteins involved in vascular reactivity, and capillary recruitment (Chikara et al., 2003; Leung et al., 2008; Rakobowchuk et al., 2008). Altogether, these adaptations would provide a favorable microvascular environment for improved microvascular reactivity, which likely explains the more sustained overshoot of StO_2_ in response ischemia/reperfusion stimulus (StO_2_ AUC) after the exercise training program in responders (Leung et al., 2008; Phillips et al., 2015).

Indeed, the contribution of the microvascular function to exercise performance has also been demonstrated. In this regard, although it has been indicated that increases in a‐vO_2diff_ after exercise training are not substantial (Skattebo et al., 2020), the skeletal muscle adaptations that ultimately contribute to changes in a‐vO_2diff_ are increases in mitochondrial volume and capillarization (Lundby et al., 2017). The contribution of the peripheral vascular adaptations to exercise capacity is also reinforced by previous findings showing a positive association between microvascular function and exercise tolerance (Alomari et al., 2004; DeSouza et al., 2000; Kingwell et al., 1996; Rainer et al., 1998). This is connected to the fact that greater microvascular responsiveness improves the ability to distribute blood flow and deliver adequate nutrients and oxygen to contracting skeletal muscle during exercise. There is also evidence showing the critical role of capillary supply to skeletal muscle in determining aerobic capacity in both humans (Hepple et al., 1997; Ingjer, 1978) and animal models (Mathieu‐Costello, 1993). Remarkably, research has shown that changes in ${\dot{V}O}_{2\max}$ occurred in parallel with changes in capillary supply (*r* = 0.63, *p* < 0.01) and capillary density (*r* = 0.44, *p* < 0.01) in a group of 20 healthy older men after aerobic training (Hepple et al., 1997). Thus, it is likely that differences in exercise adaptations between responders and non‐responders may be in part explained by the lack of positive adaptations within the lower leg microcirculation in the non‐responders.

### 4.3 Metabolic and cardiac

Regarding other metabolic and cardiac outcomes, our results showed that no exercise training‐related statistical differences were observed in body composition, blood pressure, fasting blood parameters (i.e., insulin, glucose, insulin resistance, and blood lipids), and resting cardiac measures between responders and non‐responders. Although some evidence suggest that an exercise training program of 2 weeks may be sufficient to observe improvements in ${\dot{V}O}_{2\max}$ (Honkala et al., 2017; Klonizakis et al., 2014), body composition (Motiani et al., 2017; Robinson et al., 2015), blood pressure (Honkala et al., 2017), as well as blood lipids and glucose metabolism (Honkala et al., 2017), some of the outcomes evaluated in this investigation might need a longer training process to be modified. Importantly, our findings suggest that short‐term adaptations in ${\dot{V}O}_{2\max}$ are not dependent on cardiac remodeling and that peripheral changes (i.e., microvascular responsiveness) might play an important role in the increase in ${\dot{V}O}_{2\max}$ within the 6 weeks of exercise training. The greater decrease in the $\Delta \text{HR}/\Delta {\dot{V}O}_{2}$ slope (an index of stroke volume and peripheral O_2_ extraction at a given metabolic rate) in the responders’ group, associated with the greater increase in StO_2_ AUC (a marker of microvascular responsiveness) suggest the role of improved vascular adaptations that facilitate O_2_ delivery to the exercising muscle in the short‐term cardiovascular adaptations to training.

The exercise training intervention in the present investigation was relatively short, which might explain the absence of some structural changes (e.g., heart morphology) (Lundby et al., 2017). Therefore, our discussion is limited to the results herein presented, and only applied to the short‐term effects of exercise training. More dose–response studies are required to investigate the minimum dose of exercise required to trigger adaptations.

## 5 LIMITATIONS

Individual responses to exercise training have shown to be more robustly analyzed through cross‐over designs, where participants act as their own controls (Hecksteden et al., 2015; Senn, 2018). Although we did not perform a cross‐over design, we took the following precautions to overcome this limitation: (i) the ROPE + HDI decision‐making strategy used has a considerable advantage over null‐hypothesis testing and magnitude‐based inference, such that statistical inferences are made through Bayesian credible intervals (Kruschke, 2018; Sainani, 2018); (ii) ${\dot{V}O}_{2\max}$ assessment is subject to random within‐subject and day‐to‐day variation, and for this reason, we considered a coefficient of variation of 5.6% around each individual $\Delta {\dot{V}O}_{2\max}$, as suggested elsewhere (Hecksteden et al., 2018), and (iii) we used a conservative value of 20% (the recommended value is 10%) of the pre‐training standard deviation as the minimal clinical relevant change in ${\dot{V}O}_{2\max}$ around the null value (i.e., zero), which then the percentage of the Bayesian credible interval within this region was calculated (Maturana et al.,). Additionally, although recent evidence shows that phase of the menstrual cycle does not seem to have an effect on submaximal and maximal outcomes, as well as on microvascular measures (Mattu et al., 2020; Williams et al., 2020), it should be acknowledged that our sample had an imbalanced number of males (*N* = 12) and females (*N* = 30).

## 6 CONCLUSION

In conclusion, our study showed that apart from the differences between responders and non‐responders in cardiorespiratory fitness (i.e., ${\dot{V}O}_{2\max}$), responders also presented a greater improvement in microvascular responsiveness. Interestingly, non‐responders had a greater improvement in cycling efficiency than responders. No differences were observed in the adaptations in body composition, blood pressure, fasting blood parameters, and resting cardiac measures. Our findings highlight how changes in ${\dot{V}O}_{2\max}$ (which is an important descriptor of the overall function of the cardiovascular system) might take place before other health‐related outcomes are modified. Therefore, there is an increasing need for personalized training prescription depending on the target clinical outcome.

## CONFLICT OF INTEREST

The authors declare no conflict of interest.

## AUTHOR CONTRIBUTIONS

AT, BM, and AMN conceived and designed the research project. FMM, PS, GE, CB, MW, and RNS conducted experiments. FMM, PS, GE, CB, RNS, and JMM analyzed the data. FMM wrote the manuscript. All authors read and approved the manuscript.

## Supporting information



Fig S1

Fig S2

Fig S3

Fig S4

Fig S5

Fig S6

Fig S7

Fig S8
