# Supplementary material for: Responders and non‐responders to aerobic exercise training: beyond the evaluation of V˙O2max
Source: Physiol Rep. 2021 Aug 19;9(16):e14951. doi: 10.14814/phy2.14951 (PMC8374384; doi:10.14814/phy2.14951)
Supplement: Supplementary file 1 — Fig S1 [file PHY2-9-e14951-s001.pdf]

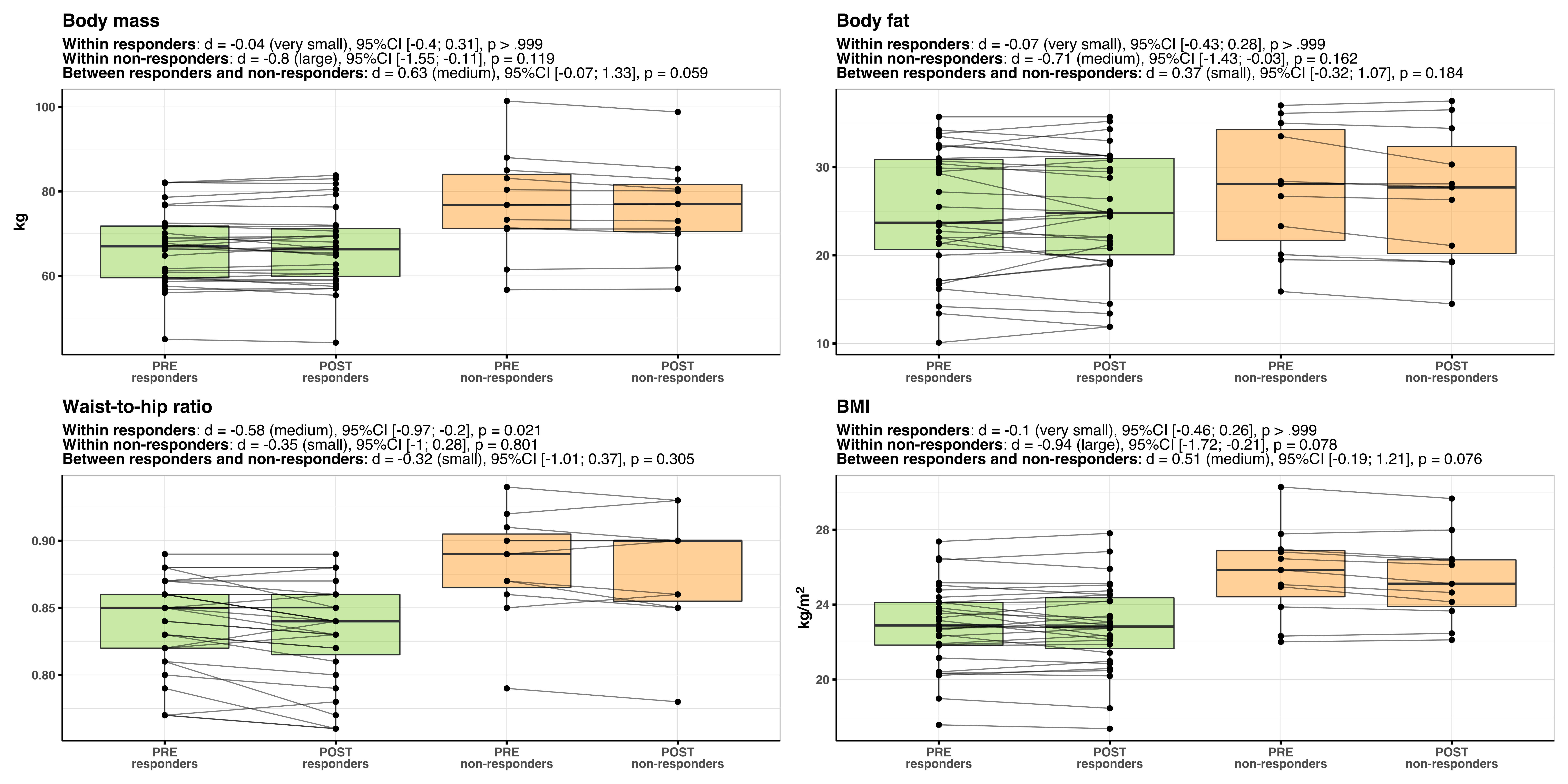
Body mass
Within responders: d = -0.04 (very small), 95%CI [-0.4; 0.31], p > .999
Within non-responders: d = -0.8 (large), 95%CI [-1.55; -0.11], p = 0.119
Between responders and non-responders: d = 0.63 (medium), 95%CI [-0.07; 1.33], p = 0.059
kg
100
80
60
PRE responders
POST responders
PRE non-responders
POST non-responders
Body fat
Within responders: d = -0.07 (very small), 95%CI [-0.43; 0.28], p > .999
Within non-responders: d = -0.71 (medium), 95%CI [-1.43; -0.03], p = 0.162
Between responders and non-responders: d = 0.37 (small), 95%CI [-0.32; 1.07], p = 0.184
30
20
10
PRE responders
POST responders
PRE non-responders
POST non-responders
Waist-to-hip ratio
Within responders: d = -0.58 (medium), 95%CI [-0.97; -0.2], p = 0.021
Within non-responders: d = -0.35 (small), 95%CI [-1; 0.28], p = 0.801
Between responders and non-responders: d = -0.32 (small), 95%CI [-1.01; 0.37], p = 0.305
0.90
0.85
0.80
PRE responders
POST responders
PRE non-responders
POST non-responders
BMI
Within responders: d = -0.1 (very small), 95%CI [-0.46; 0.26], p > .999
Within non-responders: d = -0.94 (large), 95%CI [-1.72; -0.21], p = 0.078
Between responders and non-responders: d = 0.51 (medium), 95%CI [-0.19; 1.21], p = 0.076
kg/m2
28
24
20
PRE responders
POST responders
PRE non-responders
POST non-responders
