# Supplementary material for: Responders and non‐responders to aerobic exercise training: beyond the evaluation of V˙O2max
Source: Physiol Rep. 2021 Aug 19;9(16):e14951. doi: 10.14814/phy2.14951 (PMC8374384; doi:10.14814/phy2.14951)
Supplement: Supplementary file 2 — Fig S2 [file PHY2-9-e14951-s007.pdf]

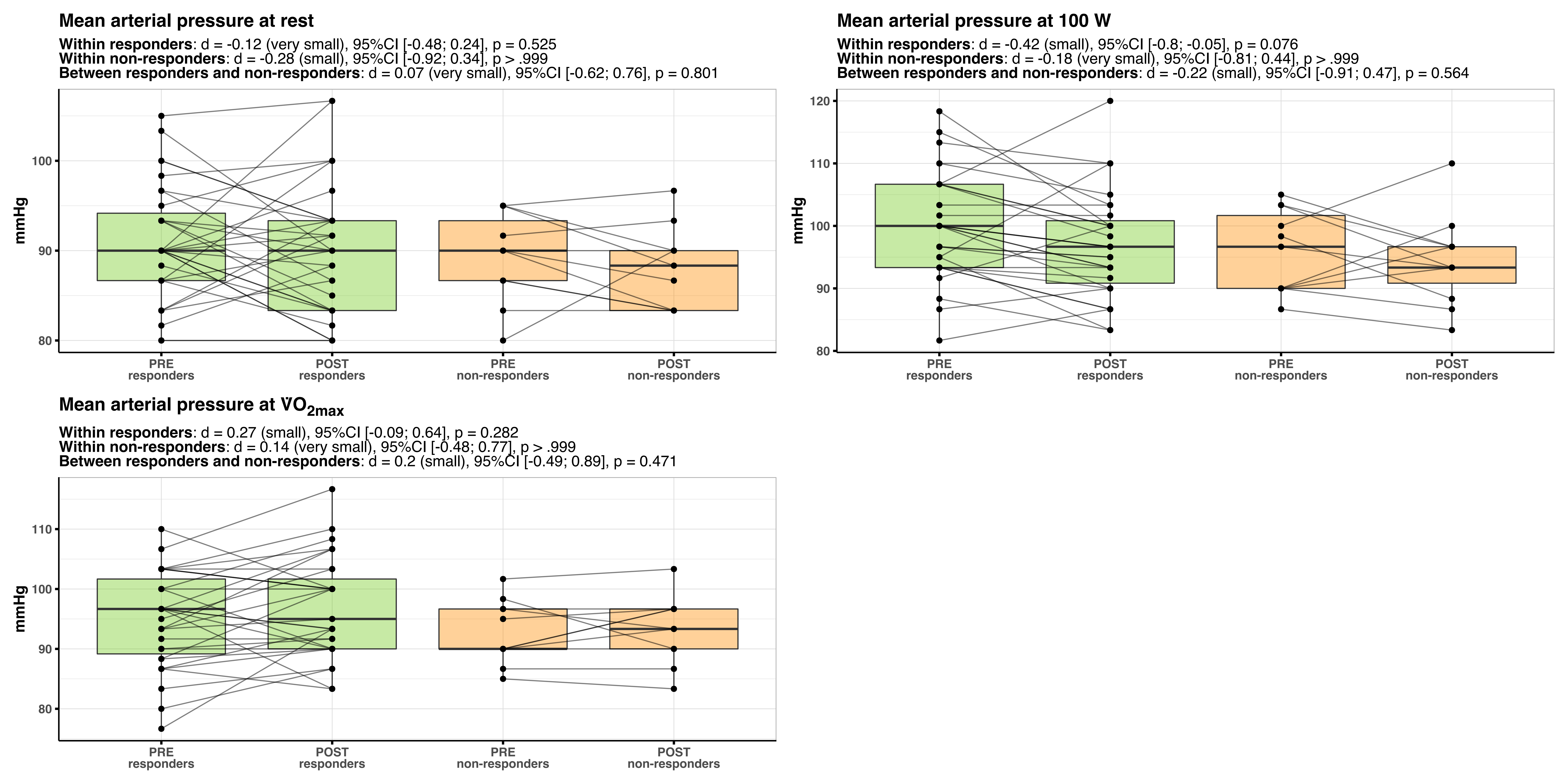
Mean arterial pressure at rest
Within responders: d = -0.12 (very small), 95%CI [-0.48; 0.24], p = 0.525
Within non-responders: d = -0.28 (small), 95%CI [-0.92; 0.34], p > .999
Between responders and non-responders: d = 0.07 (very small), 95%CI [-0.62; 0.76], p = 0.801
mmHg
80
90
100
PRE responders
POST responders
PRE non-responders
POST non-responders
Mean arterial pressure at 100 W
Within responders: d = -0.42 (small), 95%CI [-0.8; -0.05], p = 0.076
Within non-responders: d = -0.18 (very small), 95%CI [-0.81; 0.44], p > .999
Between responders and non-responders: d = -0.22 (small), 95%CI [-0.91; 0.47], p = 0.564
mmHg
80
90
100
110
120
PRE responders
POST responders
PRE non-responders
POST non-responders
Mean arterial pressure at V̇O2max
Within responders: d = 0.27 (small), 95%CI [-0.09; 0.64], p = 0.282
Within non-responders: d = 0.14 (very small), 95%CI [-0.48; 0.77], p > .999
Between responders and non-responders: d = 0.2 (small), 95%CI [-0.49; 0.89], p = 0.471
mmHg
80
90
100
110
PRE responders
POST responders
PRE non-responders
POST non-responders
