# Supplementary material for: Responders and non‐responders to aerobic exercise training: beyond the evaluation of V˙O2max
Source: Physiol Rep. 2021 Aug 19;9(16):e14951. doi: 10.14814/phy2.14951 (PMC8374384; doi:10.14814/phy2.14951)
Supplement: Supplementary file 3 — Fig S3 [file PHY2-9-e14951-s008.pdf]

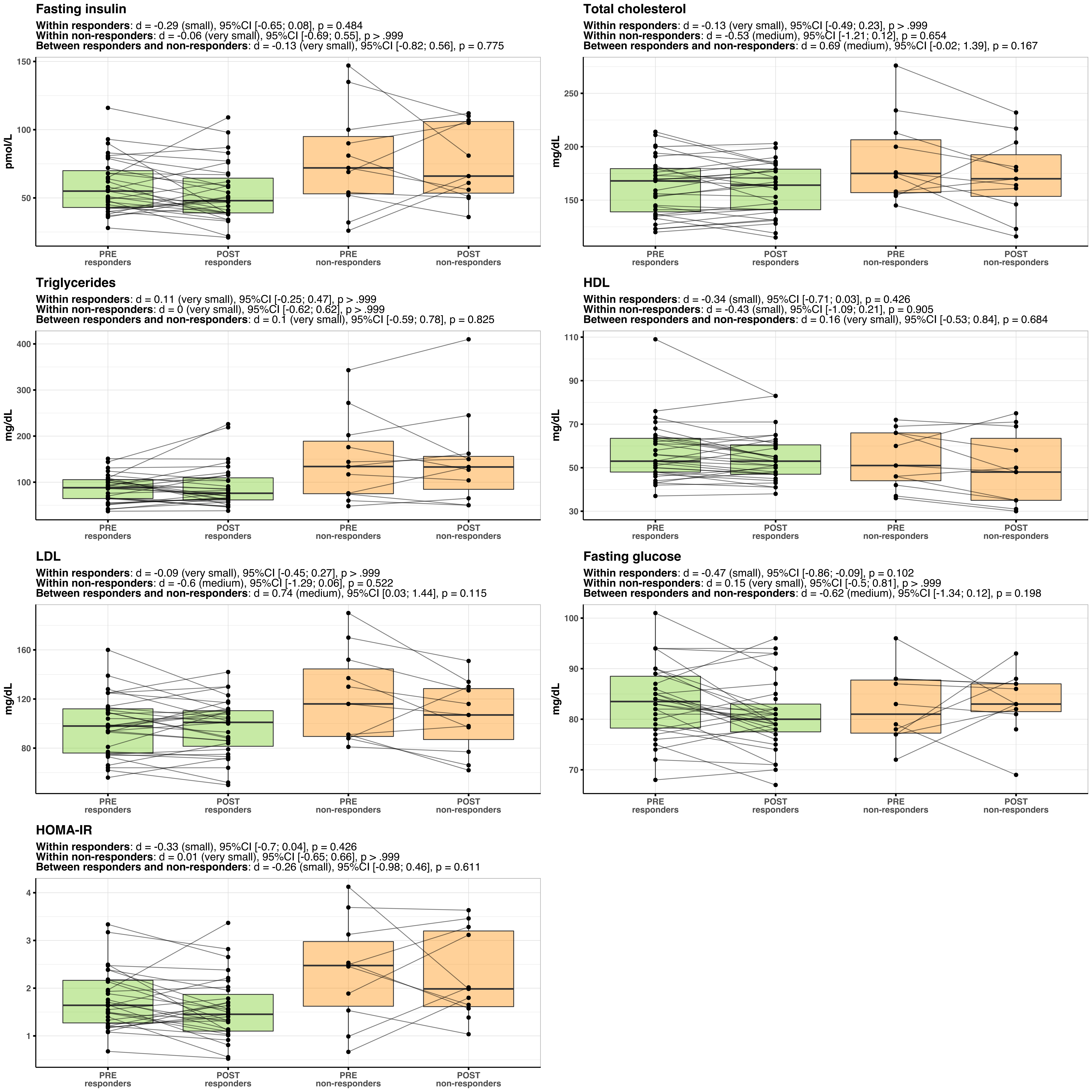
Fasting insulin
Within responders: d = -0.29 (small), 95%CI [-0.65; 0.08], p = 0.484
Within non-responders: d = -0.06 (very small), 95%CI [-0.69; 0.55], p > .999
Between responders and non-responders: d = -0.13 (very small), 95%CI [-0.82; 0.56], p = 0.775
pmol/L
Total cholesterol
Within responders: d = -0.13 (very small), 95%CI [-0.49; 0.23], p > .999
Within non-responders: d = -0.53 (medium), 95%CI [-1.21; 0.12], p = 0.654
Between responders and non-responders: d = 0.69 (medium), 95%CI [-0.02; 1.39], p = 0.167
mg/dL
Triglycerides
Within responders: d = 0.11 (very small), 95%CI [-0.25; 0.47], p > .999
Within non-responders: d = 0 (very small), 95%CI [-0.62; 0.62], p > .999
Between responders and non-responders: d = 0.1 (very small), 95%CI [-0.59; 0.78], p = 0.825
mg/dL
HDL
Within responders: d = -0.34 (small), 95%CI [-0.71; 0.03], p = 0.426
Within non-responders: d = -0.43 (small), 95%CI [-1.09; 0.21], p = 0.905
Between responders and non-responders: d = 0.16 (very small), 95%CI [-0.53; 0.84], p = 0.684
mg/dL
LDL
Within responders: d = -0.09 (very small), 95%CI [-0.45; 0.27], p > .999
Within non-responders: d = -0.6 (medium), 95%CI [-1.29; 0.06], p = 0.522
Between responders and non-responders: d = 0.74 (medium), 95%CI [0.03; 1.44], p = 0.115
mg/dL
Fasting glucose
Within responders: d = -0.47 (small), 95%CI [-0.86; -0.09], p = 0.102
Within non-responders: d = 0.15 (very small), 95%CI [-0.5; 0.81], p > .999
Between responders and non-responders: d = -0.62 (medium), 95%CI [-1.34; 0.12], p = 0.198
mg/dL
HOMA-IR
Within responders: d = -0.33 (small), 95%CI [-0.7; 0.04], p = 0.426
Within non-responders: d = 0.01 (very small), 95%CI [-0.65; 0.66], p > .999
Between responders and non-responders: d = -0.26 (small), 95%CI [-0.98; 0.46], p = 0.611
PRE responders
POST responders
PRE non-responders
POST non-responders
