# Supplementary material for: Responders and non‐responders to aerobic exercise training: beyond the evaluation of V˙O2max
Source: Physiol Rep. 2021 Aug 19;9(16):e14951. doi: 10.14814/phy2.14951 (PMC8374384; doi:10.14814/phy2.14951)

LVEDD

Within responders: d = 0.24 (small), 95%CI [−0.14; 0.61], p > .999
Within non−responders: d = −0.12 (very small), 95%CI [−0.82; 0.57], p > .999
Between responders and non−responders: d = 0.37 (small), 95%CI [−0.39; 1.12], p = 0.508

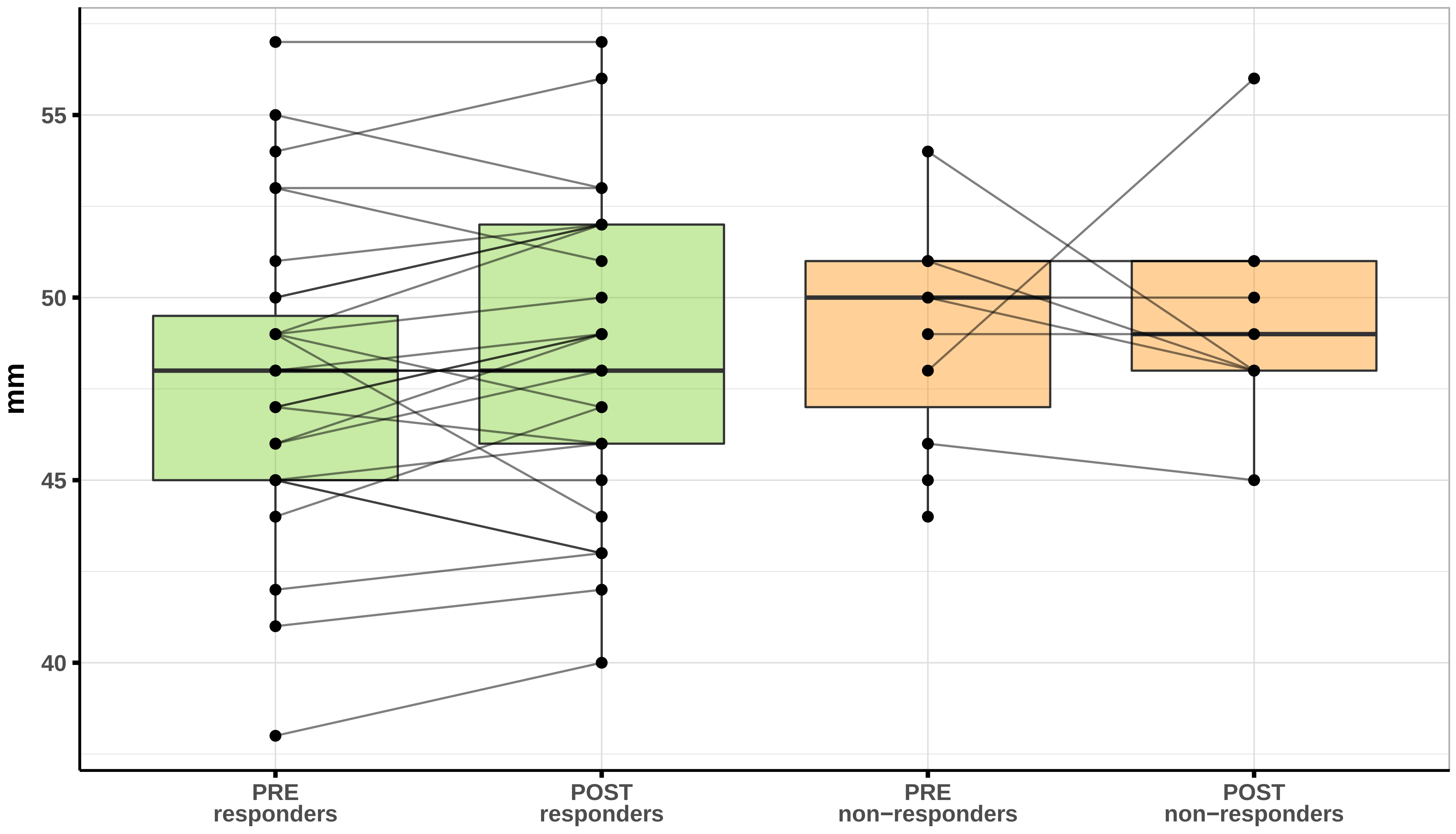

LV−Mass

Within responders: d = 0.03 (very small), 95%CI [−0.34; 0.4], p > .999
Within non−responders: d = 0.35 (small), 95%CI [−0.35; 1.08], p > .999
Between responders and non−responders: d = −0.33 (small), 95%CI [−1.08; 0.43], p = 0.412

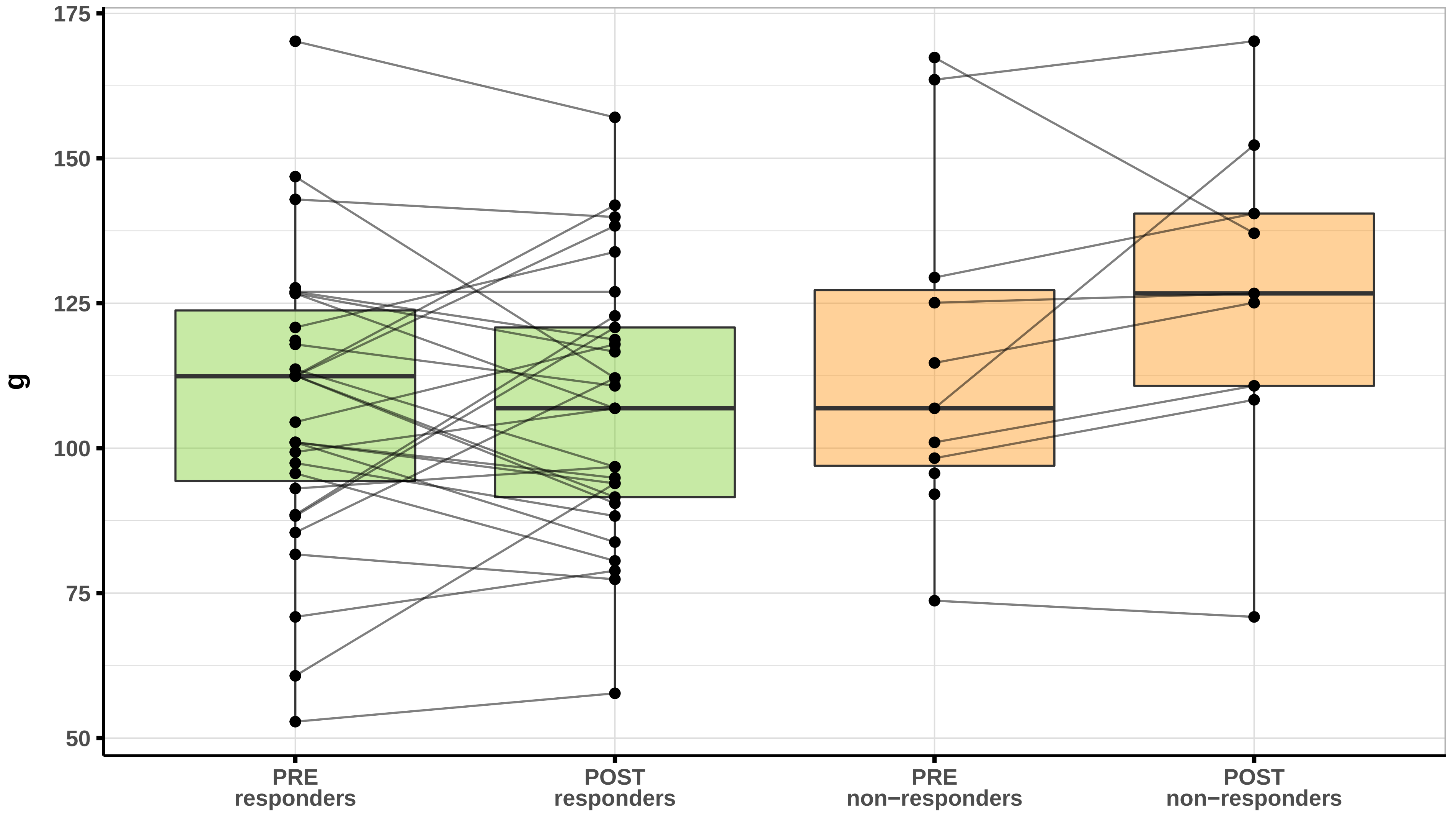

LVMI

Within responders: d = 0.03 (very small), 95%CI [−0.34; 0.4], p > .999
Within non−responders: d = 0.41 (small), 95%CI [−0.3; 1.15], p > .999
Between responders and non−responders: d = −0.36 (small), 95%CI [−1.11; 0.39], p = 0.340

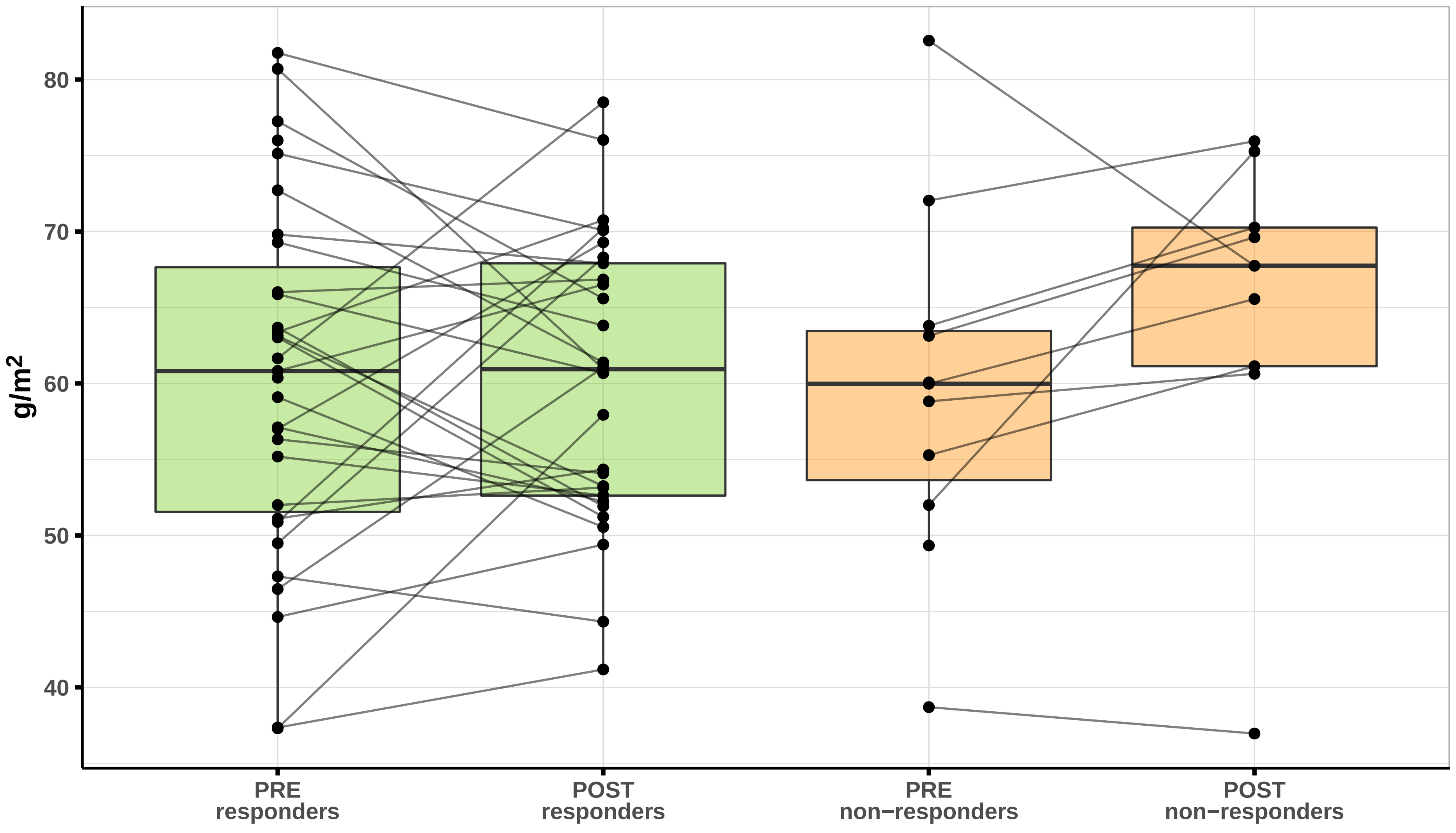

Heart volume

Within responders: d = 0.23 (small), 95%CI [−0.15; 0.6], p > .999
Within non−responders: d = −0.22 (small), 95%CI [−0.93; 0.47], p > .999
Between responders and non−responders: d = 0.42 (small), 95%CI [−0.34; 1.17], p = 0.228

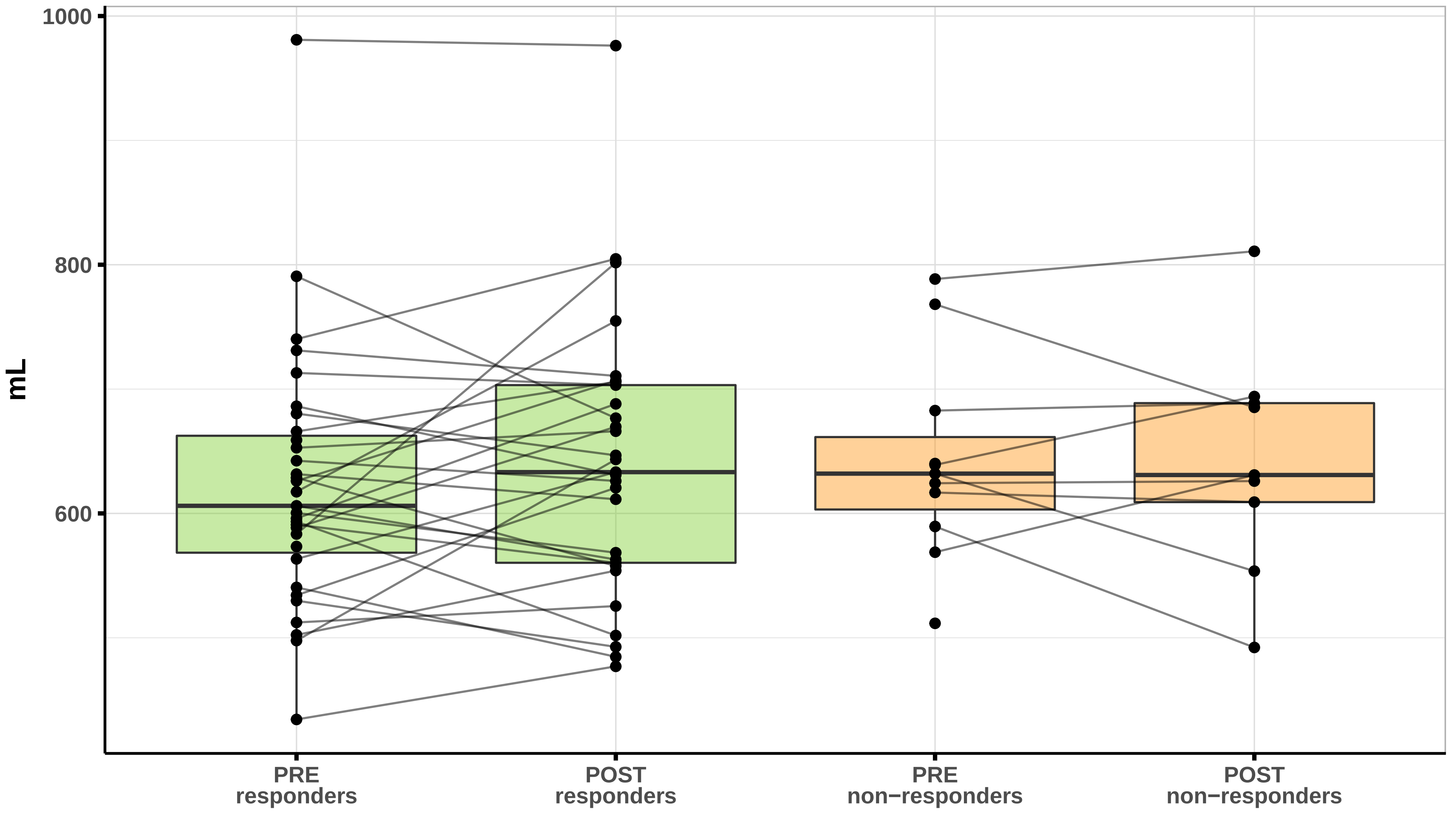

LA M−Mode

Within responders: d = 0.34 (small), 95%CI [−0.04; 0.72], p = 0.480
Within non−responders: d = −0.45 (small), 95%CI [−1.19; 0.27], p > .999
Between responders and non−responders: d = 0.77 (medium), 95%CI [0; 1.53], p = 0.057

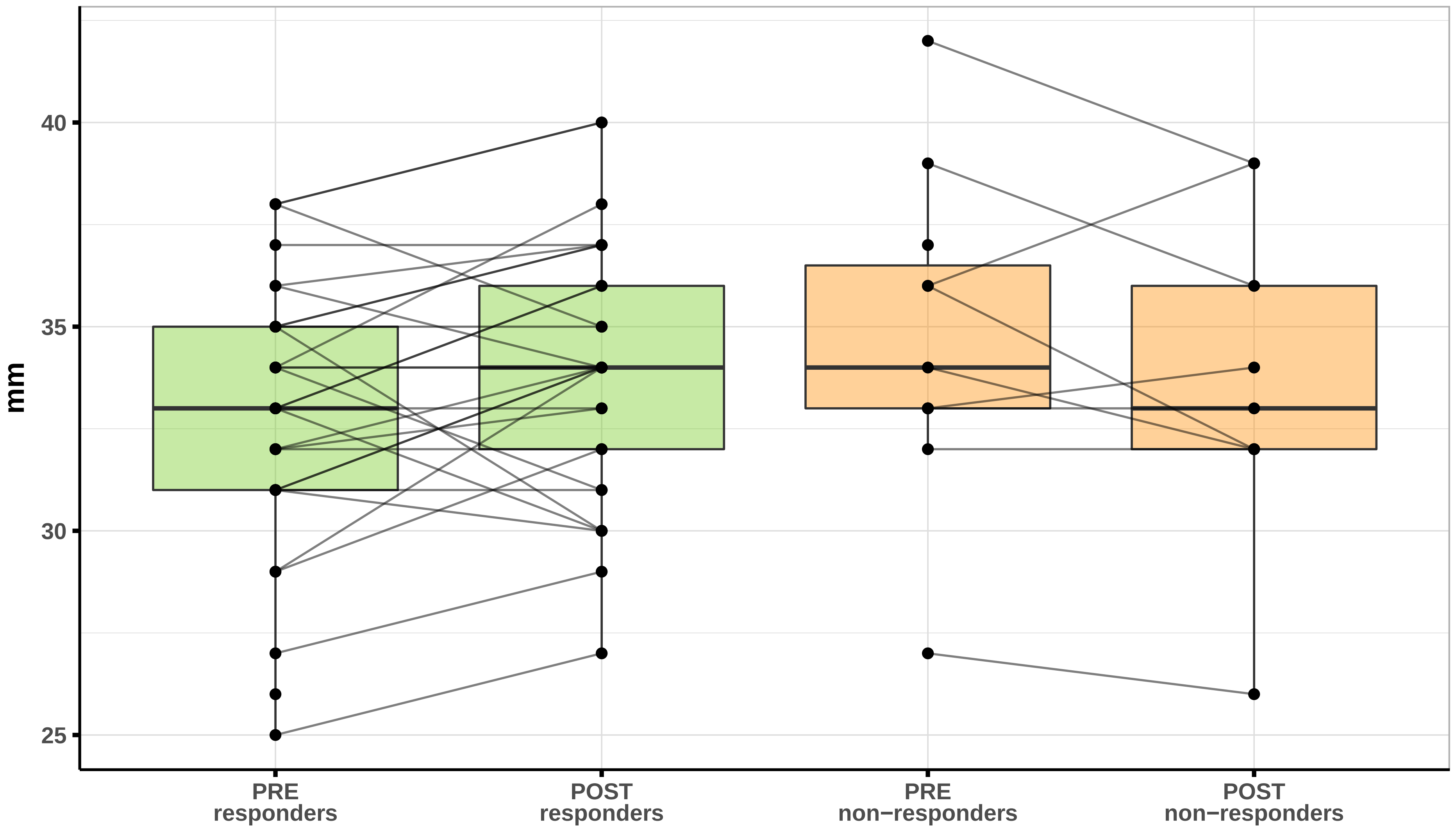

LA planimetric

Within responders: d = 0.05 (very small), 95%CI [−0.32; 0.42], p > .999
Within non−responders: d = −0.31 (small), 95%CI [−1.03; 0.39], p > .999
Between responders and non−responders: d = 0.38 (small), 95%CI [−0.38; 1.13], p = 0.362

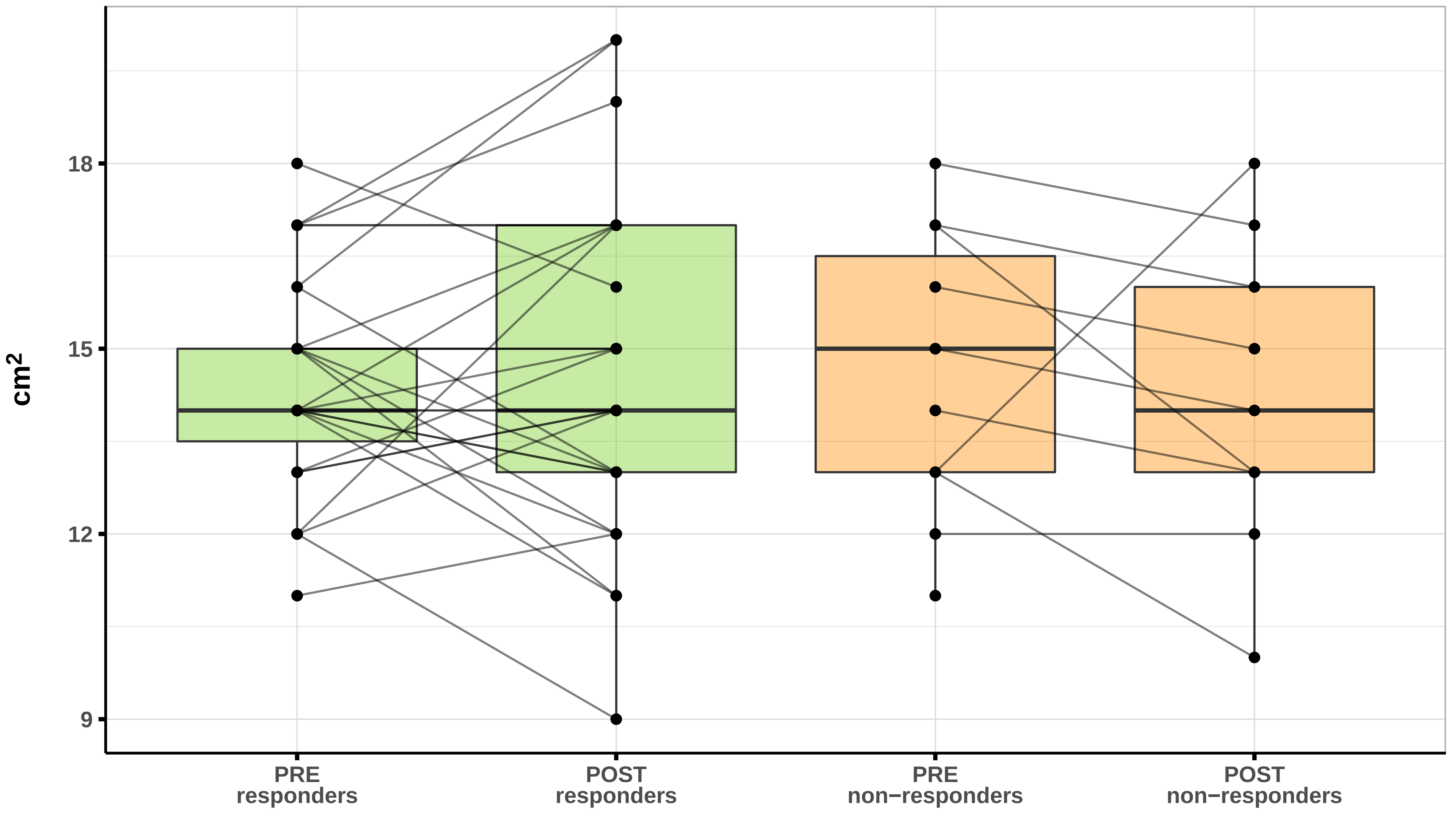

Supplement: Supplementary file 4 — Fig S4 [file PHY2-9-e14951-s006.pdf]
