# Supplementary material for: Responders and non‐responders to aerobic exercise training: beyond the evaluation of V˙O2max
Source: Physiol Rep. 2021 Aug 19;9(16):e14951. doi: 10.14814/phy2.14951 (PMC8374384; doi:10.14814/phy2.14951)
Supplement: Supplementary file 5 — Fig S5 [file PHY2-9-e14951-s002.pdf]

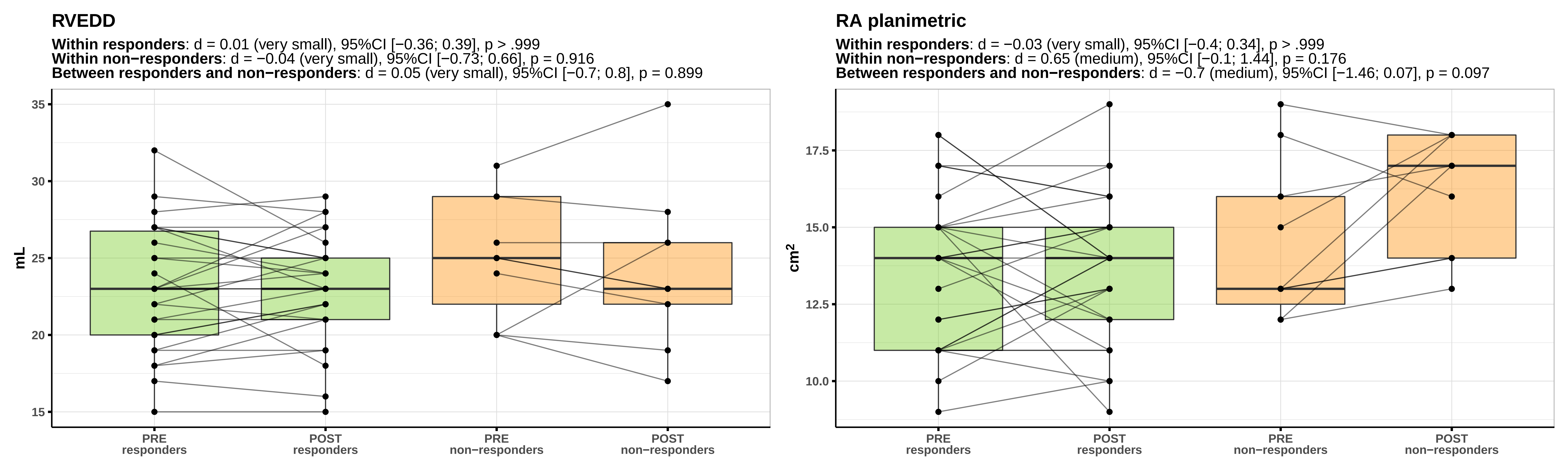

RVEDD
Within responders: d = 0.01 (very small), 95%CI [−0.36; 0.39], p > .999
Within non−responders: d = −0.04 (very small), 95%CI [−0.73; 0.66], p = 0.916
Between responders and non−responders: d = 0.05 (very small), 95%CI [−0.7; 0.8], p = 0.899
mL
35
30
25
20
15
PRE responders
POST responders
PRE non−responders
POST non−responders
RA planimetric
Within responders: d = −0.03 (very small), 95%CI [−0.4; 0.34], p > .999
Within non−responders: d = 0.65 (medium), 95%CI [−0.1; 1.44], p = 0.176
Between responders and non−responders: d = −0.7 (medium), 95%CI [−1.46; 0.07], p = 0.097
cm²
17.5
15.0
12.5
10.0
PRE responders
POST responders
PRE non−responders
POST non−responders
