# Supplementary material for: Responders and non‐responders to aerobic exercise training: beyond the evaluation of V˙O2max
Source: Physiol Rep. 2021 Aug 19;9(16):e14951. doi: 10.14814/phy2.14951 (PMC8374384; doi:10.14814/phy2.14951)
Supplement: Supplementary file 6 — Fig S6 [file PHY2-9-e14951-s005.pdf]

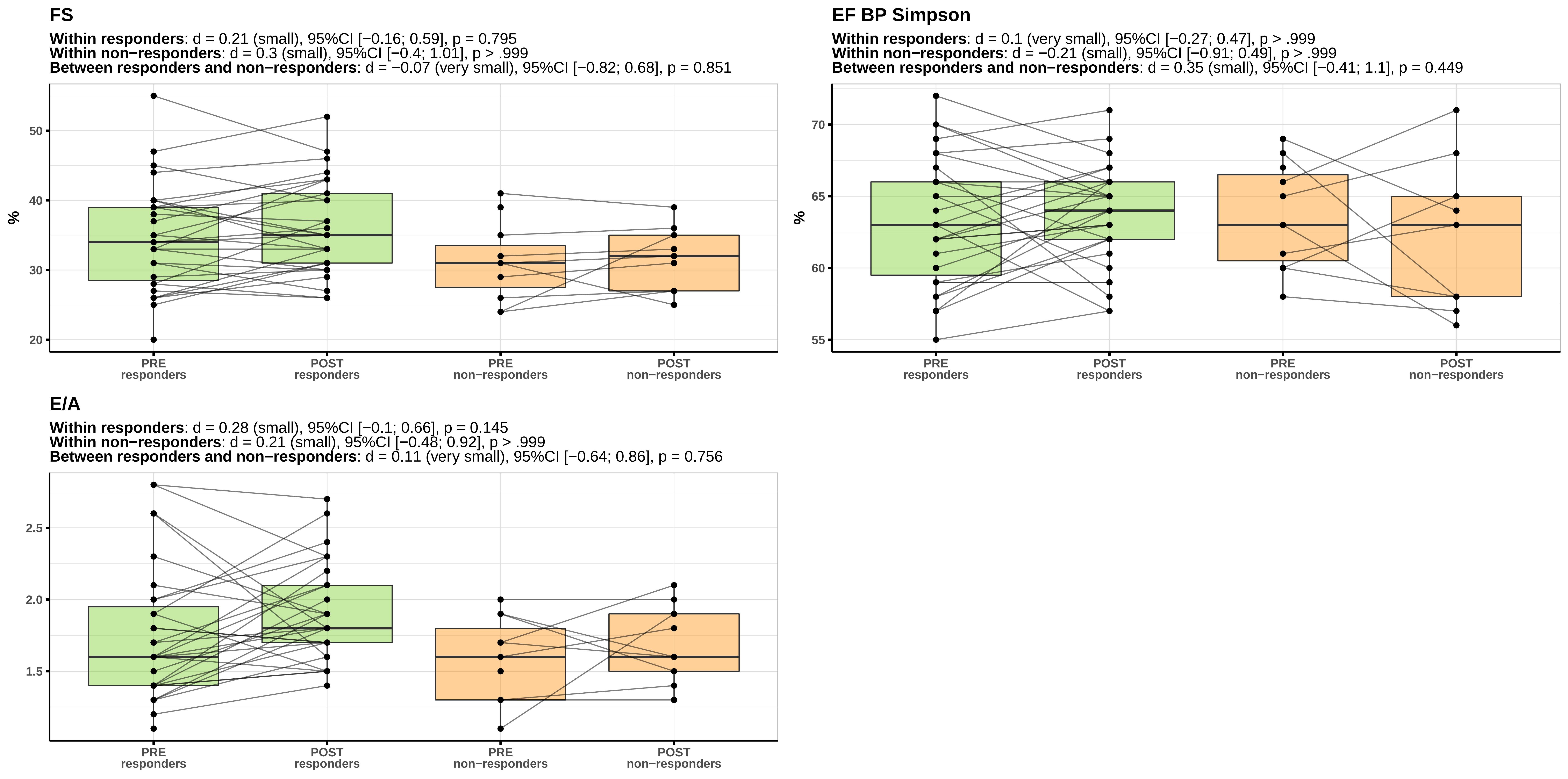
FS
Within responders: d = 0.21 (small), 95%CI [−0.16; 0.59], p = 0.795
Within non−responders: d = 0.3 (small), 95%CI [−0.4; 1.01], p > .999
Between responders and non−responders: d = −0.07 (very small), 95%CI [−0.82; 0.68], p = 0.851
%
20
30
40
50
PRE responders
POST responders
PRE non−responders
POST non−responders
EF BP Simpson
Within responders: d = 0.1 (very small), 95%CI [−0.27; 0.47], p > .999
Within non−responders: d = −0.21 (small), 95%CI [−0.91; 0.49], p > .999
Between responders and non−responders: d = 0.35 (small), 95%CI [−0.41; 1.1], p = 0.449
%
55
60
65
70
PRE responders
POST responders
PRE non−responders
POST non−responders
E/A
Within responders: d = 0.28 (small), 95%CI [−0.1; 0.66], p = 0.145
Within non−responders: d = 0.21 (small), 95%CI [−0.48; 0.92], p > .999
Between responders and non−responders: d = 0.11 (very small), 95%CI [−0.64; 0.86], p = 0.756
1.5
2.0
2.5
PRE responders
POST responders
PRE non−responders
POST non−responders
