# Supplementary material for: Responders and non‐responders to aerobic exercise training: beyond the evaluation of V˙O2max
Source: Physiol Rep. 2021 Aug 19;9(16):e14951. doi: 10.14814/phy2.14951 (PMC8374384; doi:10.14814/phy2.14951)
Supplement: Supplementary file 7 — Fig S7 [file PHY2-9-e14951-s004.pdf]

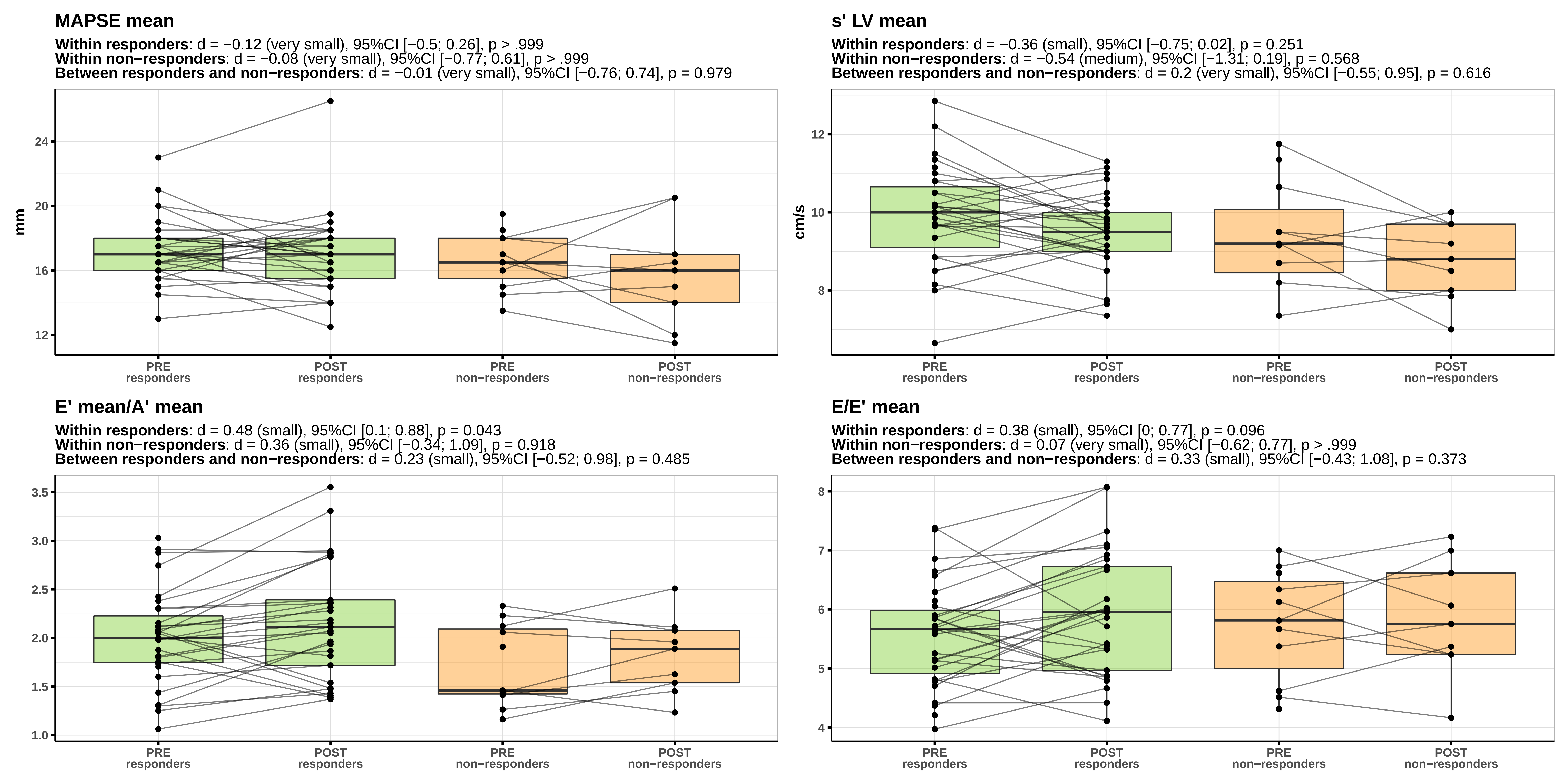
MAPSE mean
Within responders: d = −0.12 (very small), 95%CI [−0.5; 0.26], p > .999
Within non−responders: d = −0.08 (very small), 95%CI [−0.77; 0.61], p > .999
Between responders and non−responders: d = −0.01 (very small), 95%CI [−0.76; 0.74], p = 0.979
mm
24
20
16
12
PRE responders
POST responders
PRE non−responders
POST non−responders
s' LV mean
Within responders: d = −0.36 (small), 95%CI [−0.75; 0.02], p = 0.251
Within non−responders: d = −0.54 (medium), 95%CI [−1.31; 0.19], p = 0.568
Between responders and non−responders: d = 0.2 (very small), 95%CI [−0.55; 0.95], p = 0.616
cm/s
12
10
8
PRE responders
POST responders
PRE non−responders
POST non−responders
E' mean/A' mean
Within responders: d = 0.48 (small), 95%CI [0.1; 0.88], p = 0.043
Within non−responders: d = 0.36 (small), 95%CI [−0.34; 1.09], p = 0.918
Between responders and non−responders: d = 0.23 (small), 95%CI [−0.52; 0.98], p = 0.485
3.5
3.0
2.5
2.0
1.5
1.0
PRE responders
POST responders
PRE non−responders
POST non−responders
E/E' mean
Within responders: d = 0.38 (small), 95%CI [0; 0.77], p = 0.096
Within non−responders: d = 0.07 (very small), 95%CI [−0.62; 0.77], p > .999
Between responders and non−responders: d = 0.33 (small), 95%CI [−0.43; 1.08], p = 0.373
8
7
6
5
4
PRE responders
POST responders
PRE non−responders
POST non−responders
