# Supplementary material for: Responders and non‐responders to aerobic exercise training: beyond the evaluation of V˙O2max
Source: Physiol Rep. 2021 Aug 19;9(16):e14951. doi: 10.14814/phy2.14951 (PMC8374384; doi:10.14814/phy2.14951)
Supplement: Supplementary file 8 — Fig S8 [file PHY2-9-e14951-s003.pdf]

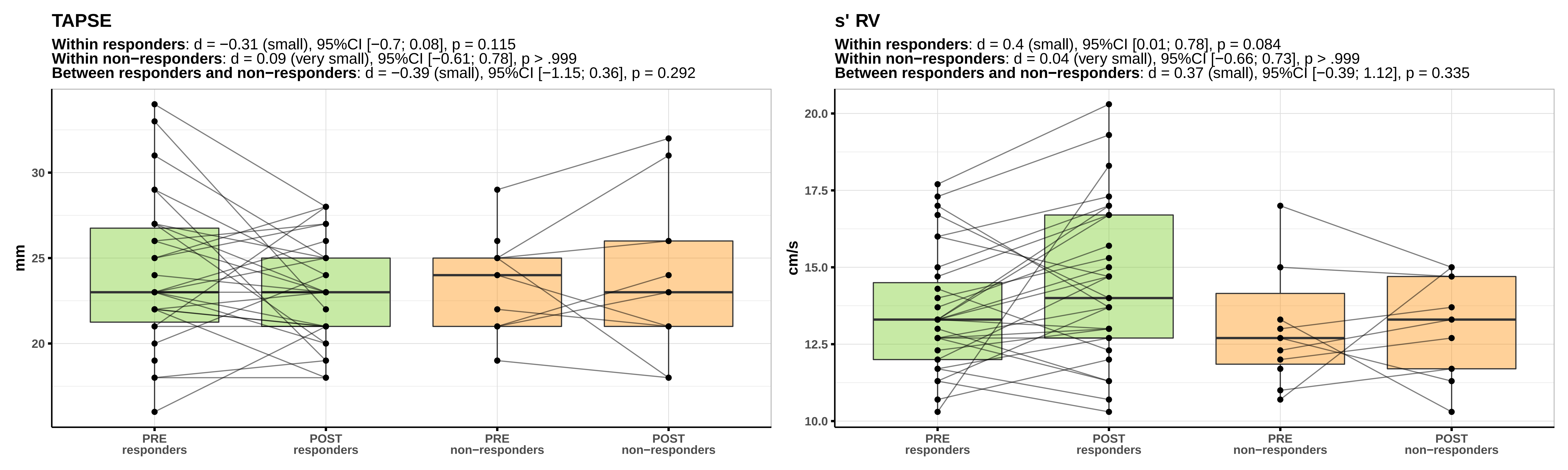
TAPSE
Within responders: d = −0.31 (small), 95%CI [−0.7; 0.08], p = 0.115
Within non−responders: d = 0.09 (very small), 95%CI [−0.61; 0.78], p > .999
Between responders and non−responders: d = −0.39 (small), 95%CI [−1.15; 0.36], p = 0.292
mm
30
25
20
PRE responders
POST responders
PRE non−responders
POST non−responders
s' RV
Within responders: d = 0.4 (small), 95%CI [0.01; 0.78], p = 0.084
Within non−responders: d = 0.04 (very small), 95%CI [−0.66; 0.73], p > .999
Between responders and non−responders: d = 0.37 (small), 95%CI [−0.39; 1.12], p = 0.335
cm/s
20.0
17.5
15.0
12.5
10.0
PRE responders
POST responders
PRE non−responders
POST non−responders
